# Widespread habitat loss and redistribution of marine top predators in a changing ocean

**DOI:** 10.1126/sciadv.adi2718

**Published:** 2023-08-09

**Authors:** Camrin D. Braun, Nerea Lezama-Ochoa, Nima Farchadi, Martin C. Arostegui, Michael Alexander, Andrew Allyn, Steven J. Bograd, Stephanie Brodie, Daniel P. Crear, Tobey H. Curtis, Elliott L. Hazen, Alex Kerney, Katherine E. Mills, Dylan Pugh, James D. Scott, Heather Welch, Riley Young-Morse, Rebecca L. Lewison

**Affiliations:** ^1^ Biology Department, Woods Hole Oceanographic Institution, Woods Hole, MA 02543, USA.; ^2^ Environmental Research Division, Southwest Fisheries Science Center, National Oceanic and Atmospheric Administration, Monterey, CA 93940, USA.; ^3^ Institute of Marine Sciences, University of California, Santa Cruz, Santa Cruz, CA 95064, USA.; ^4^ Institute for Ecological Monitoring and Management, San Diego State University, San Diego, CA 92182, USA.; ^5^ NOAA Earth System Research Laboratory, Boulder, CO 80305, USA.; ^6^ Gulf of Maine Research Institute, Portland, ME 04101, USA.; ^7^ ECS Federal, in Support of National Marine Fisheries Service, Atlantic Highly Migratory Species Management Division, Silver Spring, MD 20910, USA.; ^8^ National Marine Fisheries Service, Atlantic Highly Migratory Species Management Division, Gloucester, MA 01930, USA.; ^9^ Cooperative Institute for Research in Environmental Sciences, University of Colorado Boulder, Boulder, CO 80309, USA.

## Abstract

The Northwest Atlantic Ocean and Gulf of Mexico are among the fastest warming ocean regions, a trend that is expected to continue through this century with far-reaching implications for marine ecosystems. We examine the distribution of 12 highly migratory top predator species using predictive models and project expected habitat changes using downscaled climate models. Our models predict widespread losses of suitable habitat for most species, concurrent with substantial northward displacement of core habitats >500 km. These changes include up to >70% loss of suitable habitat area for some commercially and ecologically important species. We also identify predicted hot spots of multi-species habitat loss focused offshore of the U.S. Southeast and Mid-Atlantic coasts. For several species, the predicted changes are already underway, which are likely to have substantial impacts on the efficacy of static regulatory frameworks used to manage highly migratory species. The ongoing and projected effects of climate change highlight the urgent need to adaptively and proactively manage dynamic marine ecosystems.

## Teaser

Models predict widespread habitat loss for a number of commercially- and ecologically-important tunas, sharks and billfish.

## INTRODUCTION

Climate-driven changes in the oceans are projected to yield an average increase of 1° to 6°C in sea surface temperatures by 2100, which is likely to have profound effects on marine ecosystems and the communities, businesses, and fisheries that rely on them (*1*–*3*). Across the globe, fisheries feed and sustain an estimated 1 to 3 billion people (*4*). Changing climate conditions are likely to affect food security and livelihoods of the billions of fishers and consumers, especially those in poor countries (*5*), who rely on fish for more than 20% of their dietary animal protein (*6*). In the United States, marine fisheries and seafood industries support more than $200 billion in economic activity and 1.83 million jobs annually (*7*). While governments and management agencies worldwide have identified climate-resilient fisheries as a top priority (*8*), meeting this objective is particularly complex in highly dynamic ecosystems. Predicted changes to future ocean conditions and concomitant redistribution of marine species will likely stress many existing, spatially static, management frameworks (*8*–*11*), highlighting the need for a readily adaptable, ecosystem-based management framework fueled by dynamic models that can account for changing species distributions.

The Northwest Atlantic (NWA) and Gulf of Mexico (GOM) are dynamic and productive ecosystems that are critical regions for highly migratory species (HMS) such as sharks, tunas, and billfishes (*12*, *13*). These ecosystems have experienced some of the world’s greatest climate change impacts (*14*), which have already contributed to redistribution of nearshore fish assemblages (*14*, *15*) and caused ecological and economic disruptions in coastal systems and communities (*14*, *16*, *17*). The NWA is already among the fastest warming regions of the global ocean (*18*), and projected future changes include rapid warming (*19*, *20*) and severe marine heatwaves (*21*). Understanding the ecological and economic impacts of expected climate-induced changes in these important regions remains a central challenge and priority despite persistent knowledge gaps in how HMS will respond. 

Using three decades of satellite, oceanographic model, and in situ biological data, we developed a suite of dynamic species distribution models (SDMs) to assess how climate change has already and will continue to impact an economically and ecologically important group of 12 highly migratory apex predators in the NWA and GOM. Contemporary ocean conditions (1993–2019) are represented by a high-resolution ocean reanalysis model, and the change between the present and future (2070–2099) is obtained from dynamically downscaled global climate model outputs. Our approach uses three global climate models [Geophysical Fluid Dynamics Laboratory ESM2M (GFDL), Institute Pierre Simon Laplace CM5A-MR (IPSL), and Hadley Center HadGEM2-CC (HadGEM); see Materials and Methods] under the representative concentration pathway 8.5 (RCP8.5) emissions scenario that have been dynamically downscaled to more specifically represent future conditions in the NWA and GOM [(*20*); fig. S1]. RCP8.5 was used as this currently serves as the benchmark scenario for the U.S. National Marine Fisheries Service, including for impact assessment under the U.S. Endangered Species Act (*7*), but future work should assess other emission scenarios (*22*) as downscaled models based on phase 6 of the Coupled Model Intercomparison Project (CMIP6) become available. We integrated these oceanographic models with >228,000 fishery-dependent presence observations across 12 species (table S1) to quantify changes in species distributions and core habitats in response to both observed and projected climate change. Models for three shark (blue, porbeagle, and shortfin mako), five tuna (albacore, bigeye, bluefin, skipjack, and yellowfin), and four billfish (sailfish, blue marlin, white marlin, and swordfish) species (table S1) were trained under ocean conditions concurrent to species observations and then predicted to monthly oceanographic conditions for two time periods: the recent past (1993–2019) and future projections (2070–2099). Species-specific model predictions were summarized annually and seasonally for each global climate model to quantify expected spatial patterns of change in predator distributions and core habitat and identify species most at risk of losing habitat under climate change. This approach should yield the range of expected potential change in ocean conditions under this emission scenario and thus provide the extent of possible species responses.

## RESULTS

**Fig. 1.**
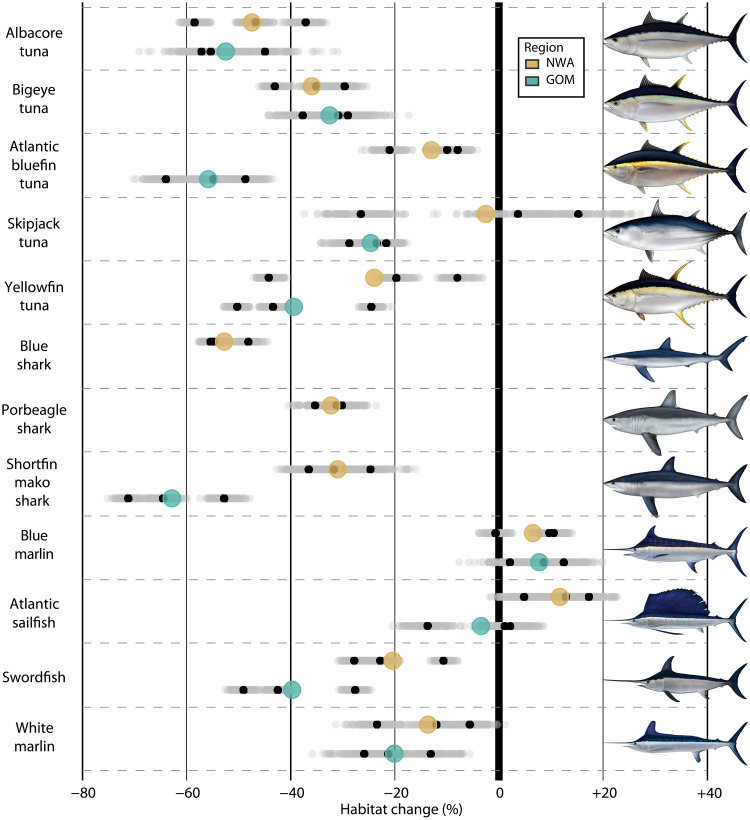
Predicted changes to top predator habitat are widespread. Overall habitat change in the Northwest Atlantic (NWA) and Gulf of Mexico (GOM). Mean of the individual climate model means is shown in color. Results for each bootstrap result (gray) and individual climate model projection mean (black) are also shown (and see fig. S14). Percentage values represent future change in suitable habitat area relative to the historical period. No results are shown for blue shark or porbeagle shark in the GOM as these species do not regularly occur in that region.

**Fig. 2.**
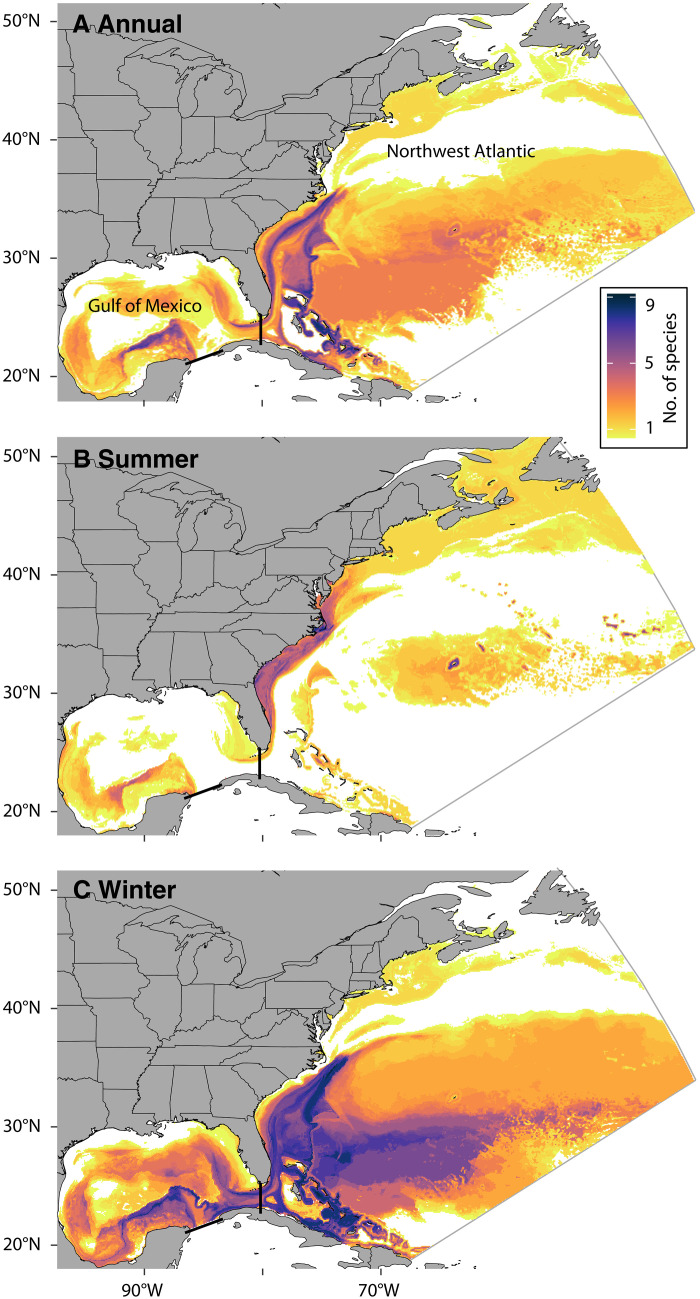
Hot spots of multi-species habitat loss are focused in the Southeast United States. Each cell represents the number of species for which habitat suitability decreased between the future and contemporary periods summarized annually (**A**) and seasonally (**B** and **C**). Only cells containing *>*20% decrease in suitability for each species are included here. Species counts are averaged over the three downscaled global climate models used to project future habitat suitability. Downscaled global climate model domain is shown in gray outline. Region boundaries for NWA and GOM are indicated in black lines. See fig. S15 for corresponding annual and seasonal gains.

We found that projected changes to the ocean environment (fig. S1) will lead to widespread loss in suitable habitat for 9 of the 12 species in the NWA (mean, −21%; range, −62% to +35%; Fig. 1 and figs. S2 to S13). Of the 10 species that regularly inhabit the GOM, our model results predicted that 8 would experience considerable habitat losses (mean, −32%; range, −75% to +9%; Fig. 1). Despite variability among species habitat projections for each of the climate models (fig. S14), increased suitable habitat was predicted for only one species—blue marlin—in both regions. The magnitude of future habitat losses ranged from 20% for swordfish and yellowfin tuna in the NWA to >60% for Atlantic bluefin tuna and shortfin mako shark in the GOM (Fig. 1). Projected declines in suitable HMS habitat were focused in the Southeast United States and southern GOM during winter where as many as nine species are expected to experience at least a 20% decrease in future habitat suitability in the same area (Fig. 2). Future gains in habitat suitability were of smaller magnitude and focused near Newfoundland during summer and in the Gulf Stream during winter (fig. S15).

**Fig. 3.**
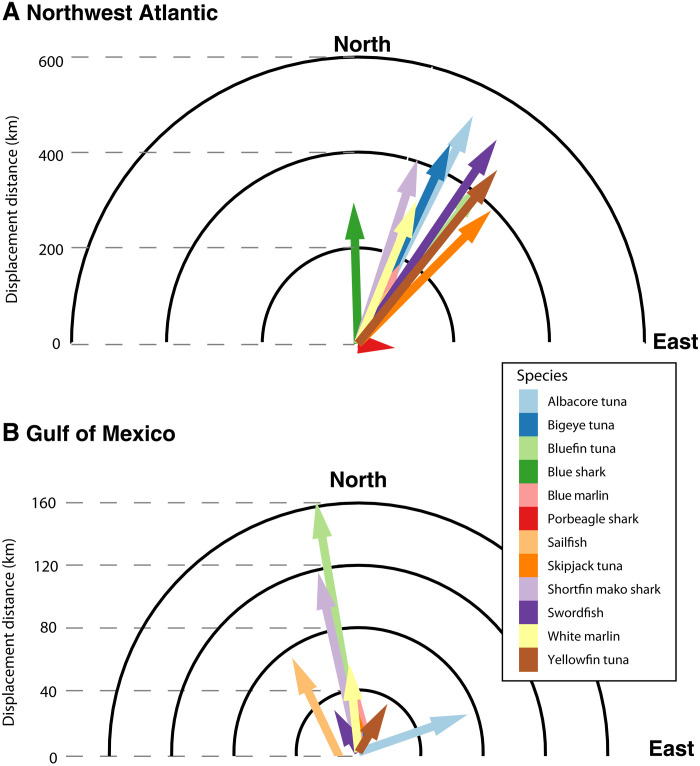
Displacement of core predator habitat is substantial and consistently northward. Mean displacement direction and distance of HMS core habitat between the contemporary (1993–2019) and future (2070–2099) periods are shown for the Northwest Atlantic (**A**) and Gulf of Mexico (**B**) across the downscaled climate models. Note the distance axes are on different scales.

These changes in habitat suitability for Atlantic HMS were linked to substantial displacement of predicted species distributions. For all species in the NWA, except porbeagle shark, large-scale changes to the overall suitability of habitat in the region resulted in displacement of core habitat north and east up to 532 km by the end of the century (mean, 354 km; range, 87 to 532 km; Fig. 3). The projected changes to species distributions in the GOM are smaller in displacement distance, and direction of change is more variable as the size and shape of the basin effectively prevent suitable habitats from moving further north as the ocean warms. Despite these geographic constraints, average expected displacement is 66 km and primarily northward (range, 31 to 163 km; Fig. 3).

**Fig. 4.**
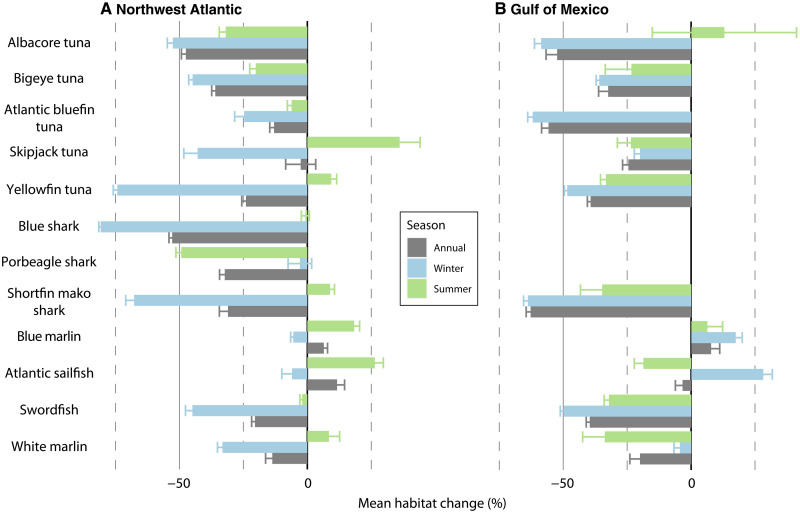
Annual mean change can mask strong seasonal variability. Overall change in habitat suitability summarized annually and by season (winter, December to February; summer, June to August) in the NWA (**A**) and GOM (**B**).

While we focus on longer-term changes represented by annual averages of predicted habitat suitability, strong seasonal signals of change were also evident from monthly model predictions (Fig. 4 and fig. S15). Our results indicate most species will experience disproportionate change in one of the two seasons analyzed here (summer: June to August, winter: December to February). For example, the annual average of habitat change for yellowfin tuna in the NWA is a 24% loss by the end of the century; however, the annual average masks the seasonal variability, which indicates that winter habitat loss is more than triple the annual mean (74%) while summer change indicates a minor increase in habitat (9%) over the same time period (Fig. 4 and fig. S6). Similarly, strong seasonal variability is apparent in the analysis of multi-species hot spots of habitat change (fig. S15). The annual average indicates concentrated areas of losses focused in the Southeast United States and virtually no areas of substantial multi-species gains. While some multi-species increases in habitat suitability are apparent, particularly in the Northeast United States and Scotian Shelf during summer, the extent and magnitude of hot spots in multi-species habitat loss are amplified during winter.

**Fig. 5.**
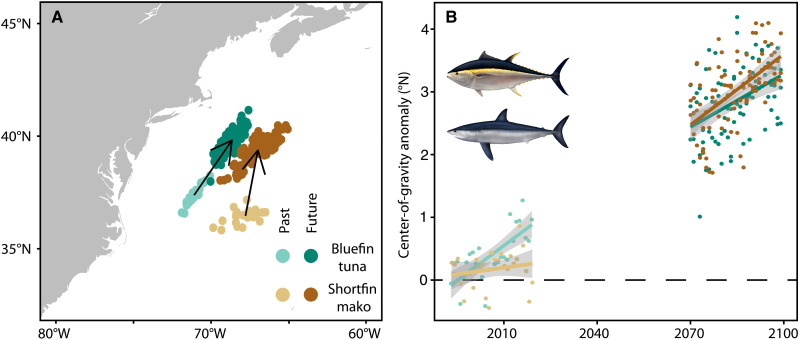
The impacts of climate change are already substantial for some species. Bluefin tuna and shortfin mako sharks are predicted to experience similar displacement in COG by the end of the century (**A**), but bluefin COG has in the last 30 years already re-distributed one-third of the expected distance (**B**). See fig. S16 for all species in both regions.

In addition to finding compelling evidence of end-of-century changes in habitat and distribution, our analyses reveal that the impacts of climate change are already observable. Our results show consistent northward displacement in the NWA for nearly all species in the recent past (1993–2019; fig. S16). For example, shortfin mako shark and bluefin tuna are both expected to experience center-of-gravity (COG) displacement *>*3° northward (*>*400 km) by the end of the century (Fig. 5); however, core habitat for Atlantic bluefin tuna has already moved one-third of this distance. Indeed, approximately 25% of expected displacement has occurred across all species in both regions (NWA: mean, 21%; range, 5 to 50%; GOM: mean, 28%; range, 6 to 51%; fig. S16). While larger changes are expected in the future, there is uncertainty in model projections at longer timescales. Our results indicate that some of the expected climate change impacts are already happening.

## DISCUSSION

Our model projections suggest that HMS in the NWA and GOM will continue to experience substantial declines in suitable habitat from ocean warming and other impacts of climate change, with most habitat losses ranging from 20 to 65% and displacement of core habitat averaging ∼350 km. The projected changes in species’ habitats were largely consistent across the three downscaled global climate models, which were selected to represent a range of ocean response to climate-induced forcing (*20*). Despite variability among climate models, this region is expected to experience widespread warming by the end of the century with enhanced sea level rise in coastal regions and higher salinity along the southeast U.S. coastline (fig. S1). These changes correspond to a substantial northward shift in pelagic biodiversity, leading to increased potential for changes in human-wildlife interactions (*23*) for these ecologically and economically important species.

The magnitude of the observed changes that have already occurred, paired with the projected changes over the coming decades, highlight critical issues regarding the ecological viability of HMS assemblages in the NWA and GOM. Previous climate research has identified climate change "winners" and "losers" (*24*–*26*), with the risk of loss predicted to be elevated for species with a limited tolerance for temperature fluctuations (*27*) or with other sensitivities that limit their ability to adapt to environmental change (*26*). Although our correlative model framework cannot account for potential species adaptability or thermal tolerance, our results suggest predominant and widespread habitat loss for nearly all HMS studied here, even though many of these species have exceptional adaptations that reduce their sensitivity to ambient water temperatures (*28*). Furthermore, as temperatures increase, existing life history strategies, such as adult bluefin spawning at or near their upper thermal limit in the GOM (*29*), may no longer be viable (*30*, *31*), although the extent to which species could seek thermal refugia at depth remains unknown.

The shifts in species habitat and distributions also raise concerns for associated fisheries (*32*, *33*) and the socioeconomic impacts of climate change on fishing communities (*2*, *34*, *35*). Our results identify hot spots for species habitat loss, concentrated in the Southeast United States and southern GOM, where substantial decreases in habitat suitability are expected for multiple species. Furthermore, our models indicate that these losses will not be offset by increased suitable habitat within our model domain for any species, although we do identify minor regions of potential multi-species gain in the northern part of the domain during summer. Many of the most substantial changes are predicted at the boundaries of species distributions. Rapid shifts at the trailing edges of species’ distributions are well documented, particularly in regions characterized by weak spatial gradients in ocean properties [e.g., (*36*)]. In our study, the combination of the importance of temperature in driving species’ habitat use (table S2), the variability in temperature gradients across the region, and the relatively constant warming expected across the whole model domain (fig. S1) likely result in more rapid change at the trailing edges of species’ distributions. This is in contrast to the leading edges where strong oceanographic gradients result in less dramatic displacement despite widespread warming.

The spatially concentrated impacts of ocean warming and species redistributions are likely to have substantial socioeconomic impacts on fishing fleets that target these regions and especially domestic fleets based in the Southeast United States, suggesting region-specific vulnerability as marine resources redistribute among regional and national jurisdictions and international waters (*5*, *37*). The magnitude of these impacts appears to be substantial: We found that seasonal effects for many species were even larger than annual change (*38*), a finding that aligns with other published studies [e.g., blue marlin; (*39*, *40*)]. Our analyses to understand how habitat and associated distributions change are also likely influenced by data availability, in this case fishery-dependent data. While recent work suggests projecting SDMs using fishery-dependent data can still result in robust inference (*41*), a similar analysis using fishery-independent (*25*) or integrative modeling approaches (*42*) would be a valuable next step toward constraining species’ response to climate change. Similarly, concurrent changes to species abundance (*38*, *43*) and predator-prey relationships (*44*) may further compound the complex responses of HMS to expected climate changes.

Concentrated changes in species distributions, as shown here, highlight the need for adaptive management approaches that can respond to expected change. For example, static fishery closure or bycatch reduction areas may need to be re-evaluated more frequently to continuously meet their management objectives (*11*, *45*, *46*). For the NWA and GOM, the spatially and temporally dynamic nature of species responses to climate change is evident. However, most existing management frameworks in these ocean areas are spatially static (e.g., time/area closures) or are linked to nation-specific catch quotas. Regardless, they are not responsive to managing highly mobile organisms that respond to shifting ocean dynamics [e.g., (*46*–*48*)] and mobile human pressures (*49*), particularly in the context of increasing anomalous ocean conditions and longer-term change to marine ecosystems (*8*, *9*). Our model framework and associated results represent an eco-informatic approach that can be used to project changing species distributions and commensurate bycatch risk, and assess the efficacy of long-standing, static spatial management frameworks in the context of changing species distributions (*11*, *45*). These models also provide the necessary ingredients for dynamic ocean management approaches that embody the ephemeral nature of the ocean environment, species, and human uses when designing management strategies (*50*, *51*). Our results suggest that static fishery management measures will continue to lose ecological relevance and economic efficacy as species redistribute under climate change. Further, when anticipated changes in species distributions are not implicit to management strategies, unexpected fisheries and protected species conflict are likely to increase (*52*, *53*). More climate-focused research and monitoring efforts are needed to design and deploy dynamic management frameworks that advance climate resilience and readiness.

## MATERIALS AND METHODS

### Species occurrence data and pseudo-absences

We used two fishery-dependent datasets to represent species occurrence (table S1). We obtained marker tag data from the International Commission for the Conservation of Atlantic Tunas (ICCAT) Secretariat tag database (https://iccat.int/en/) for our target species in the Atlantic Ocean from 1959 to 2019. These marker (e.g., conventional or "spaghetti") tags are attached to a fish upon release and may be recorded again if the individual is later recaptured. Recaptures comprised 13% of the observations in this dataset and were treated as independent from the release observations. Three gear types comprised the majority of marker tag observations, including 58% from rod and reel, 17% from longline, and 14% from unclassified gear codes. We also used catch data from an at-sea observer program that monitors the U.S. Atlantic pelagic longline fishery, which has been in place since the early 1990s. In this program, independent observers catalog gear and catch information for every set made on ∼10 to 15% of longline fishing trips (*46*, *54*). These observer data were used to represent catch of individual fish through the spatial extent of the fishery concentrated in the northern GOM, along the east coast of the United States and along the southern and eastern edges of the Grand Banks. Data were filtered to include the time period for which environmental data were available from the oceanographic model (1993–2019, see below). For both datasets, records were removed if the date or coordinates contained errors that could not be resolved, if observations were duplicated, or when coordinates indicated the position was on land or outside the Atlantic Ocean.

A fundamental challenge of many data types for habitat modeling is that they are presence-only, and thus cannot provide information on animal absence. A number of techniques have been developed to simulate data representing where individuals were likely absent, often termed "pseudo-absences" (*55*–*57*). Here, we used pseudo-absences for both occurrence datasets, including in place of "true" absences in the fishery observer data because recent work found that pseudo-absences resulted in substantial improvements in ecological realism of SDM predictions compared to the same models trained with absences from the fishery observer data (*58*). Thus, we generated pseudo-absences for the pooled, species-specific observations using background sampling methods by randomly drawing without replacement from the monthly spatial extent of the full species-specific dataset (*56*, *58*). The spatial extent for pseudo-absence sampling was defined using a minimum convex polygon that contained monthly (all years), species-specific occurrences. Dates were assigned to pseudo-absence locations by randomly drawing from the possible dates in the corresponding species-presence dataset. Pseudo-absences were compared against all available presence data to avoid generating pseudo-absences for which there was a corresponding presence observation. To reduce any autocorrelation structure in the data and to provide independence between data points, we thinned all presence and pseudo-absence observations to ensure only one event occurred in that month (regardless of year) and 0.1° grid cell (∼10 km) following (*59*). Resulting pseudo-absence locations were randomly sub-sampled to generate a 1:1 presence/pseudo-absence ratio for each species dataset, which has been recommended for boosted regression tree (BRT) modeling approaches (*55*).

### Oceanographic data and models

We included five environmental variables as potential predictor variables in the SDMs, which consisted of two static variables and three dynamic surface variables. The dynamic environmental data were sourced from the Global Ocean Physics Reanalysis [“GLORYS”, Copernicus Marine Environmental Monitoring Service; (*60*)]. GLORYS is a global, data-assimilating ocean model with daily outputs at 1/12° (∼9 km) horizontal resolution representing 50 vertical levels. The data assimilating nature of the model allows for regular data-driven updates to model predictions from in situ platforms and remote sensing observations that ensure realistic model outputs. The three dynamic surface variables included the following: (i) sea surface temperature (in degrees Celsius); (ii) sea surface height (in meters); and (iii) sea surface salinity (in practical salinity units). These variables were chosen based on availability in both the GLORYS model and the downscaled global climate models (see below). The two static variables included bathymetry (ETOPO1 obtained from https://ngdc.noaa.gov/mgg/global/global.html, coarsened to 1/12°; in meters) and rugosity (calculated as the spatial standard deviation of bathymetry over a 0.25° square; in meters). Each corresponding environmental value was extracted from the presence/pseudo-absence locations and times for each data type and included in the final data frame that was used as training data for each species-specific SDM. All environmental grids used the GLORYS native spatial (1/12°) and temporal (monthly) resolution.

Future oceanographic conditions were represented by Regional Ocean Model System [ROMS; (*61*, *62*)] simulations of the NWA Ocean described in (*20*). The model, which extends from the GOM to Newfoundland, has a 7-km horizontal resolution and 40 vertical levels. Alexander *et al.* (*20*) conducted a ROMS control simulation for the period 1976–2005 and three ROMS simulations representing future conditions under the RCP8.5 scenario from the CMIP5 archive. Surface and side boundary conditions were obtained from the NOAA GFDL, IPSL, and HadGEM global climate models. These models represented a range of climate responses to anthropogenic forcing as indicated by weak, moderate, and strong response in global surface temperature in the GFDL, IPSL, and HadGEM models, respectively. The future values were derived using the "delta method" in which the 30-year monthly mean values for 1976–2005 and 2070–2099 were computed separately for the three climate models and then the differences between the period means were added to the boundary conditions used in the control run. We scaled the ROMS response to the delta forcing, obtained from the future simulations minus the control, from those originally reported by Alexander *et al.* (*20*) to account for the more recent historical period represented by GLORYS (1993–2019, as used here) compared to the original control period (1976–2005). The ocean changes relative to the 1976–2005 climate are adjusted by a factor of 0.9094, so they are relative to the 1993–2019 period. The scaling is obtained from the ratio of the change in total greenhouse gas forcing between 2070–2099 and 1993–2019 divided by the change in forcing between 2070–2099 and 1976–2005. Brickman *et al.* (*63*) and Siedlecki *et al.* (*64*) used this method to determine the scaling values for 2050 and 2100. Year-to-year variability in the response is retained during the 2070–2099 period and the total values are obtained by adding the scaled response to the GLORYS climatology. ROMS "delta" outputs were re-projected to match the GLORYS grid using bivariate interpolation methods from the akima package for R (*65*) and custom implementation code (*66*) and added to the GLORYS climatology.

### Species distribution models

Habitat suitability was modeled for each species as a function of environmental variables using a BRT framework [dismo R package, (*67*)]. BRTs are non-parametric and use boosting (a numerical optimization technique) to determine optimal partitioning of variance. One of the advantages of using BRTs is their ability to handle correlation and collinearity effects of the environmental variables so a priori assessment of predictor variables is not needed (*67*). BRTs for each species were fitted using a Bernoulli family appropriate to the binary nature of the response variable (presence/pseudo-absence) and a fixed number of 2000 trees with a learning rate of 0.005, a bag fraction of 0.75, and a tree complexity of 5 [for a thorough discussion of hyper-parameter tuning, see (*68*)]. The resulting models describe species-specific “habitat suitability” as continuous values ranging from 0 to 1.

Model training data were sourced from the full North Atlantic extent of the study region (100 to 5°W, 10 to 55°N) and pooled between data types for each species. Data pooling was used to combine inference across observation datasets as models trained on pooled data have been shown to be effective for combining inferences across data types and provide more ecologically realistic predictions than individual, data-specific models (*58*).

Models were assessed using 10-fold cross-validation where each dataset was randomly split into 75% training and 25% testing data (*69*). A model was fit on the training data and used to explain and predict habitat suitability in the testing dataset. At each cross-validation step, we assessed model explanatory power and predictive skill. Explanatory power indicates a model’s ability to explain the variability in a given dataset and was evaluated using percent explained deviance (*R*^2^). Predictive skill indicates how well a model prediction can discern different actual outcomes (*70*) and was evaluated with area under the receiver operating characteristic curve (AUC), true skill statistic (TSS), and balanced accuracy, a metric that integrates sensitivity and specificity (*71*). Model assessment values are presented in table S1, and variable importance values are reported in table S2.

### Data analysis

Fitted models were predicted to monthly outputs for the base period (GLORYS, 1993–2019) and future climate scenarios (each of the three ROMS simulations, 2070–2099). Predictions were averaged annually and over summer [JJA (June, July, August)] and winter [DJF (December, January, February)] seasons within individual years to quantify expected changes in habitat suitability between contemporary and future ocean conditions. Thresholds used to define species-specific core habitats were derived from model fits following (*72*). The thresholds were calculated by converting each monthly prediction during the historical period into binary presence/absence and using an algorithm from the pROC package for R that optimizes the proportion of these predictions that are correctly assigned to presence/absence data records for each species (*73*, *74*). The mean of these species-specific, monthly thresholds was used as the threshold to classify core habitat for each species from monthly habitat suitability predictions for both the historical and future periods across the study region.

Given the different basin shape and morphology, as well as potential species redistribution under climate change, the remaining metrics were calculated for the NWA and GOM separately. To capture shifts in species distributions and habitat in response to climate change, we calculated three metrics: COG, relative change in habitat, and multi-species habitat gains and losses. Classified core habitat for each species was used to calculate the COG of species’ monthly distributions using the SDMTools package for R (*75*), which describes the spatial mean of an animal’s core habitat area for that time period. COG displacement between the historical and future periods across ROMS simulations was calculated by fitting a linear regression to derived COG locations per year within each time period at two temporal scales: annual and sub-annual (summer and winter seasons). Given the interannual variability in predictions for some species, the fitted regressions were used to predict start and end COGs for each period (e.g., annual COG at the start and end of GLORYS period). These summarized COGs were used to calculate expected future displacement distance and direction using rdist.earth.vec and bearing functions in the fields (*76*) and geosphere (*77*) packages for R. COG displacements were considered northward (positive) if the bearing of displacement was between 0° and 90° or between 270° and 360°, and southward (negative) if the bearing was 90° to 270°. Relative change in suitable habitat area, as percentage of historical habitat suitability, was calculated by comparing mean habitat area for each species in the last decade of the future period (2090–2099) minus historical mean habitat area (1993–2002), divided by the historical mean habitat area (1993–2002). Grid cells with species-specific habitat suitability gains or losses greater than 20% were used for the multi-species "hot spot" analysis. This threshold was used to highlight only those species for which a relatively strong, and likely ecologically meaningful, gain or loss signal (*>*20% change) was predicted. Each ROMS simulation was compared to GLORYS to generate a mean number of species that are expected to gain or lose according to >20% change in habitat suitability between historical and future periods. We present the mean across the three ROMS simulations. To generate summary statistics, including confidence intervals, for all derived metrics, we used bootstrapping (*n* = 100) in which models were re-fit to 75% of the original training data at each bootstrap iteration. Each fit model (100 iterations for each of 12 species = 1200 models) was predicted to monthly oceanographic fields for the historical (GLORYS; *n* = 324) and future (*n* = 360 months × 3 ROMS simulations = 1080) periods.

## Supplementary Materials

### This PDF file includes:

Figs. S1 to S16 Tables S1 and S2 References
