# Supplementary material for: Widespread habitat loss and redistribution of marine top predators in a changing ocean
Source: Sci Adv. 2023 Aug 9;9(32):eadi2718. doi: 10.1126/sciadv.adi2718 (PMC10411898; doi:10.1126/sciadv.adi2718)
Supplement: Supplementary file 1 — Figs. S1 to S16 Tables S1 and S2 References [file sciadv.adi2718_sm.pdf]

Science Advances
AAAS

Supplementary Materials for

Widespread habitat loss and redistribution of marine top predators in a changing ocean

Camrin D. Braun *et al.*

Corresponding author: Camrin D. Braun, cbraun@whoi.edu

Sci. Adv. **9**, eadi2718 (2023)
DOI: 10.1126/sciadv.adi2718

This PDF file includes:

Figs. S1 to S16
Tables S1 and S2
References

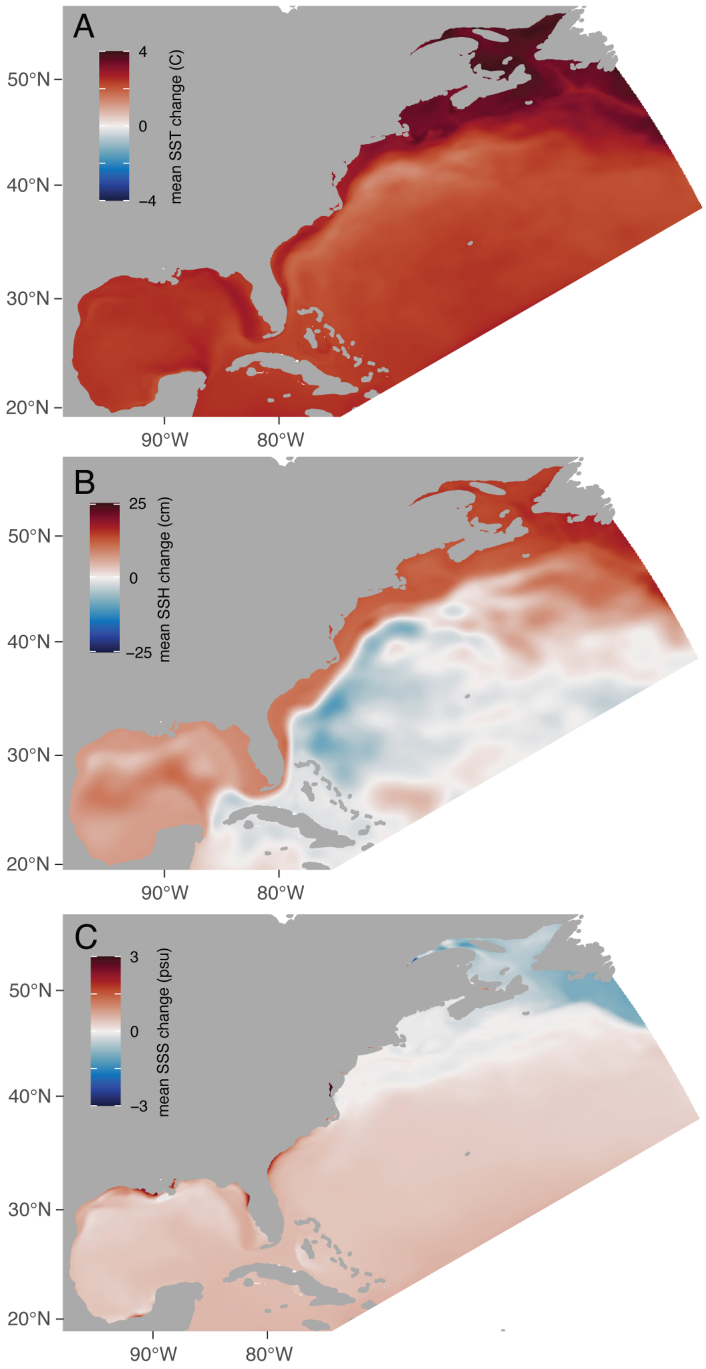

Figure S1: **Models predict significant changes to ocean conditions.** Mean expected future ocean conditions from three downscaled global climate model simulations (CMIP5) under the business as usual emissions scenario RCP8.5 from (20). Panels show expected change ("delta") in a) sea surface temperature, b) sea surface height and c) sea surface salinity.

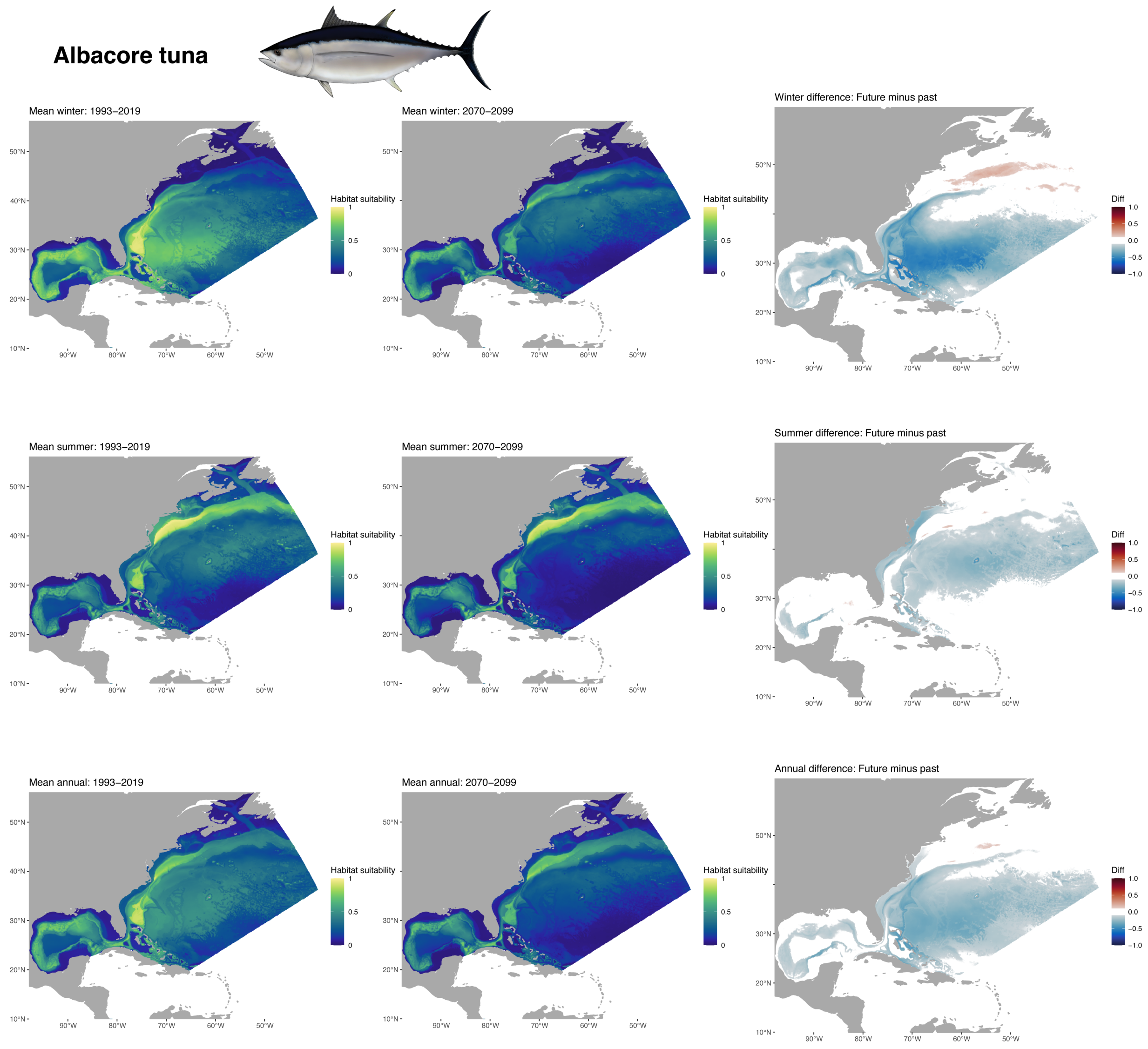

Figure S2: **Predicted change in species-specific habitat demonstrate significant spatial and seasonal variability.** Model predicted habitat suitability for albacore tuna during summer (top row), winter (middle row) and the annual average (bottom row) for the current (left column) and future periods (middle column) and the expected change (right column). These predictions use the GFDL downscaled climate model. Note that ±10% change in habitat suitability in the right column is masked (white) to improve visual clarity for more substantial expected changes.

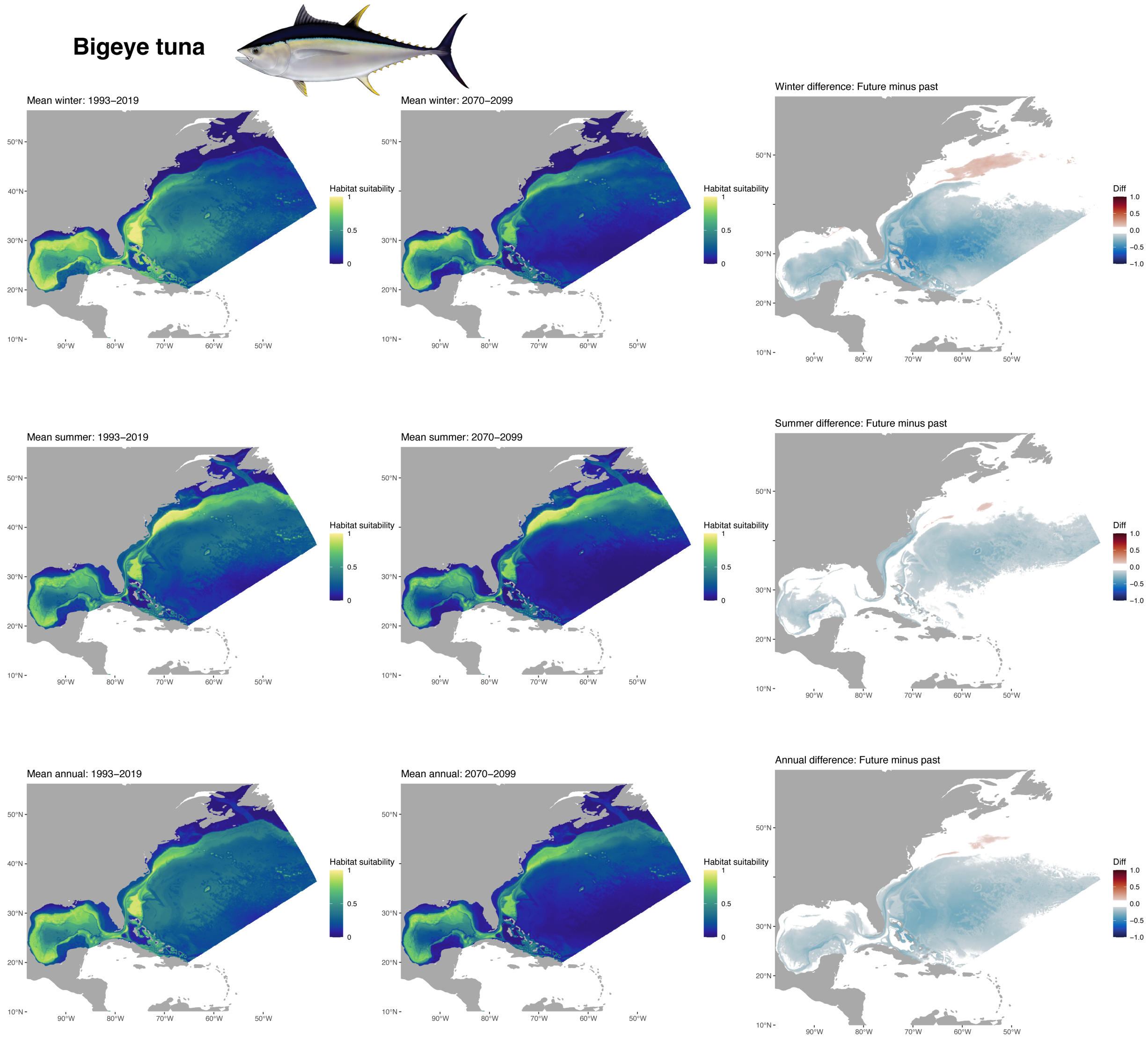

Figure S3: **Predicted change in species-specific habitat demonstrate significant spatial and seasonal variability.** Model predicted habitat suitability for bigeye tuna during summer (top row), winter (middle row) and the annual average (bottom row) for the current (left column) and future periods (middle column) and the expected change (right column). These predictions use the GFDL downscaled climate model. Note that ±10% change in habitat suitability in the right column is masked (white) to improve visual clarity for more substantial expected changes.

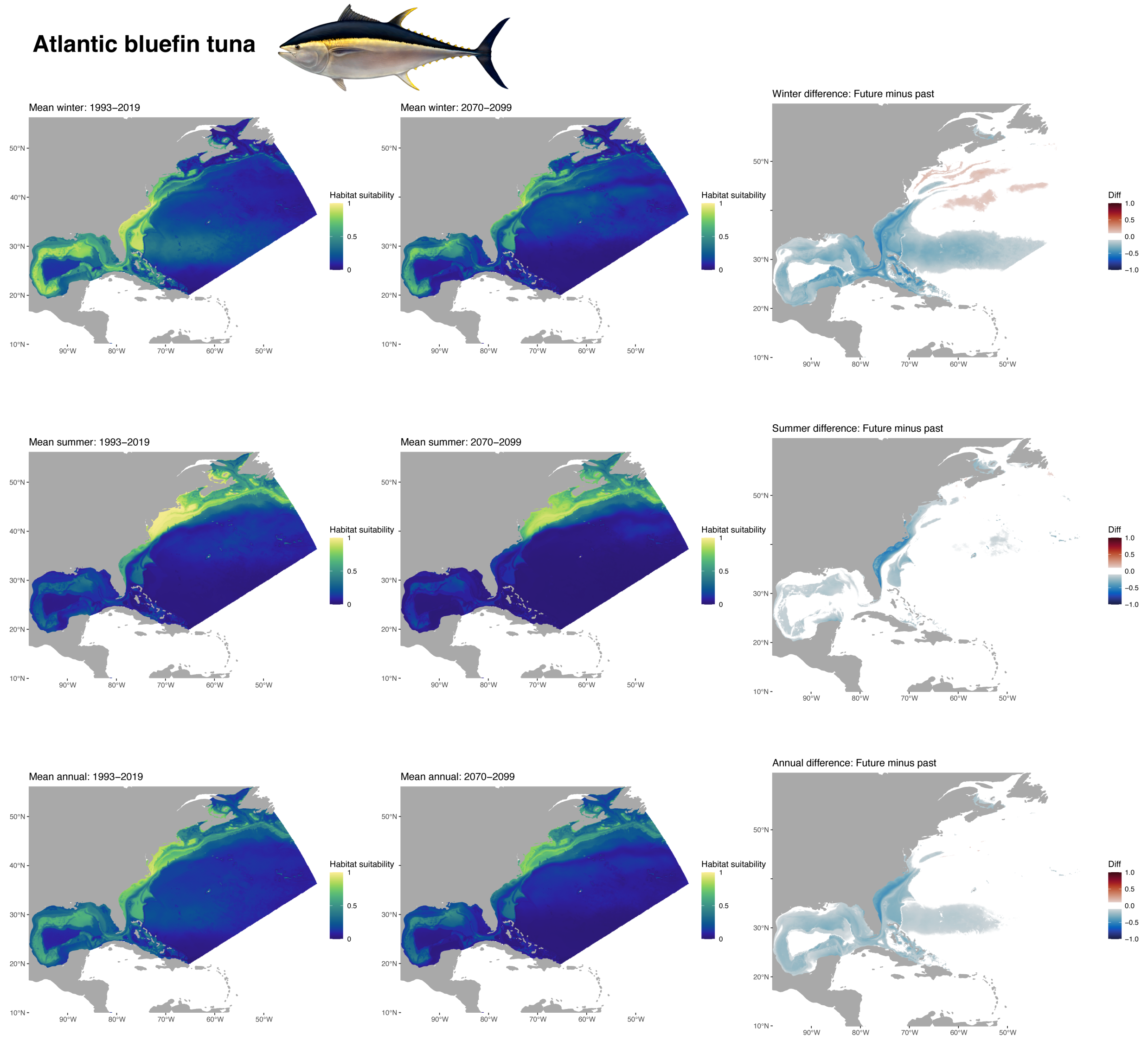

Figure S4: **Predicted change in species-specific habitat demonstrate significant spatial and seasonal variability.** Model predicted habitat suitability for Atlantic bluefin tuna during summer (top row), winter (middle row) and the annual average (bottom row) for the current (left column) and future periods (middle column) and the expected change (right column). These predictions use the GFDL downscaled climate model. Note that ±10% change in habitat suitability in the right column is masked (white) to improve visual clarity for more substantial expected changes.

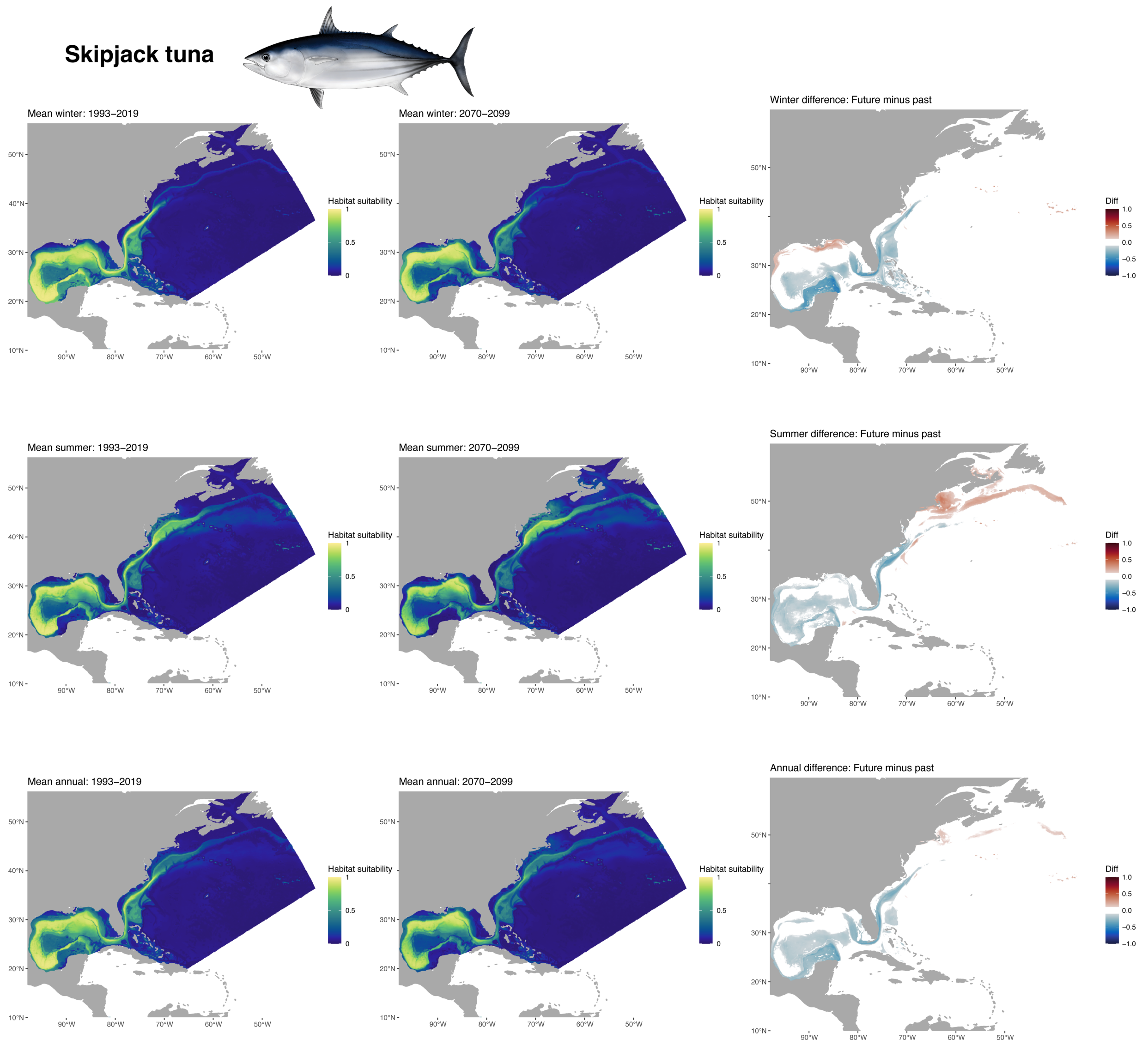

Figure S5: **Predicted change in species-specific habitat demonstrate significant spatial and seasonal variability.** Model predicted habitat suitability for skipjack tuna during summer (top row), winter (middle row) and the annual average (bottom row) for the current (left column) and future periods (middle column) and the expected change (right column). These predictions use the GFDL downscaled climate model. Note that ±10% change in habitat suitability in the right column is masked (white) to improve visual clarity for more substantial expected changes.

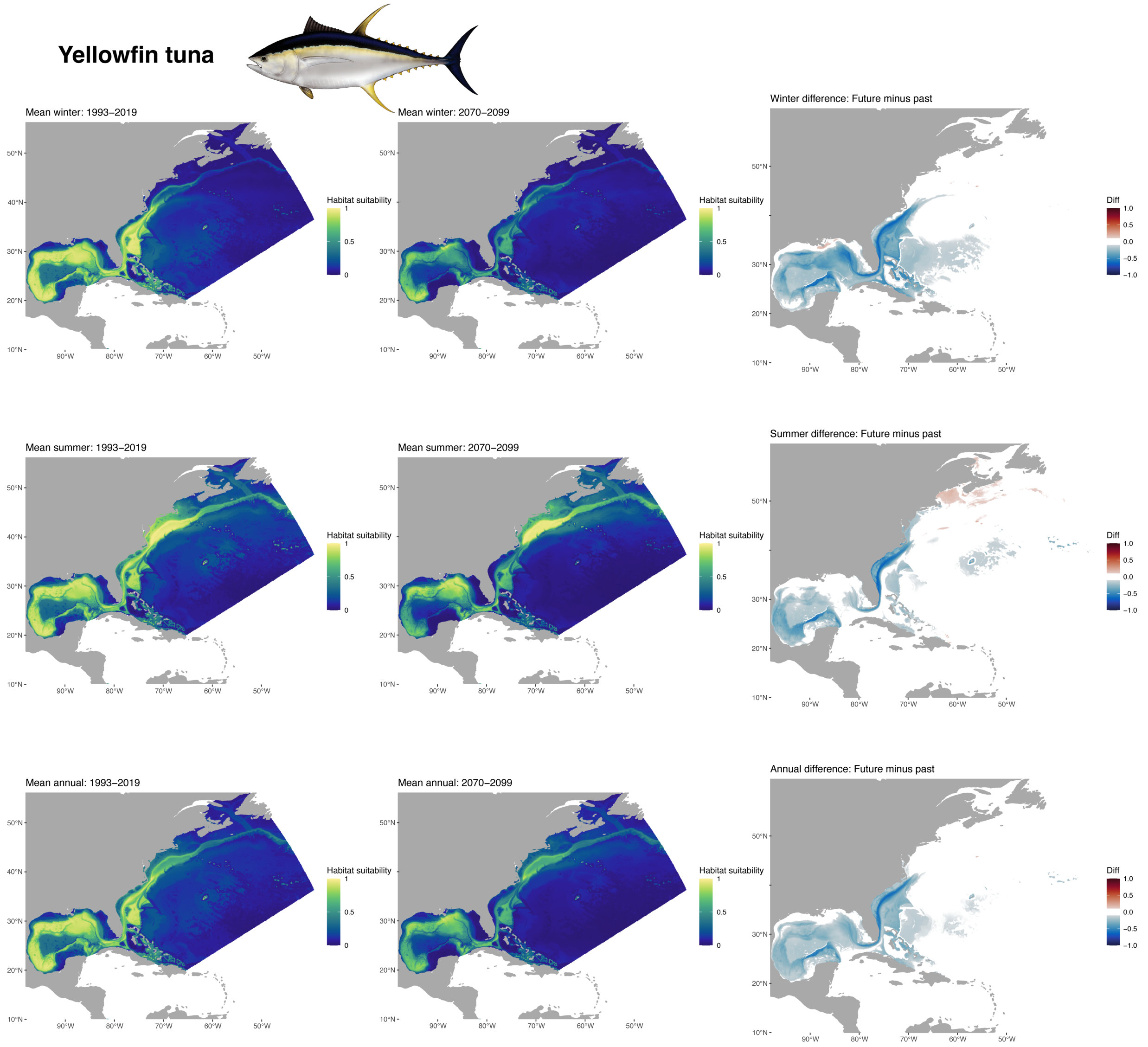

Figure S6: **Predicted change in species-specific habitat demonstrate significant spatial and seasonal variability.** Model predicted habitat suitability for yellowfin tuna during summer (top row), winter (middle row) and the annual average (bottom row) for the current (left column) and future periods (middle column) and the expected change (right column). These predictions use the GFDL downscaled climate model. Note that ±10% change in habitat suitability in the right column is masked (white) to improve visual clarity for more substantial expected changes.

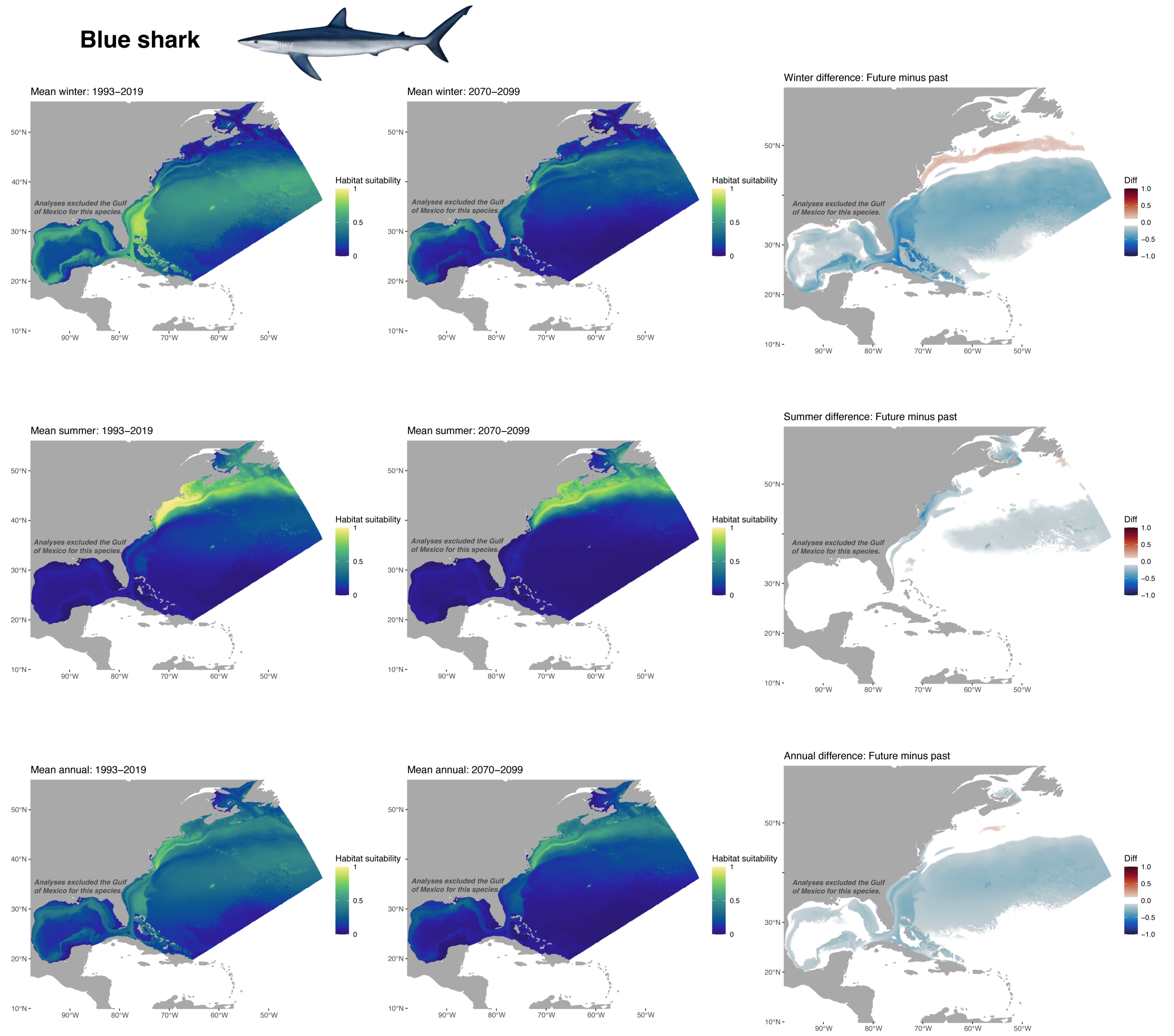

Figure S7: **Predicted change in species-specific habitat demonstrate significant spatial and seasonal variability.** Model predicted habitat suitability for blue shark during summer (top row), winter (middle row) and the annual average (bottom row) for the current (left column) and future periods (middle column) and the expected change (right column). These predictions use the GFDL downscaled climate model. Note that ±10% change in habitat suitability in the right column is masked (white) to improve visual clarity for more substantial expected changes.

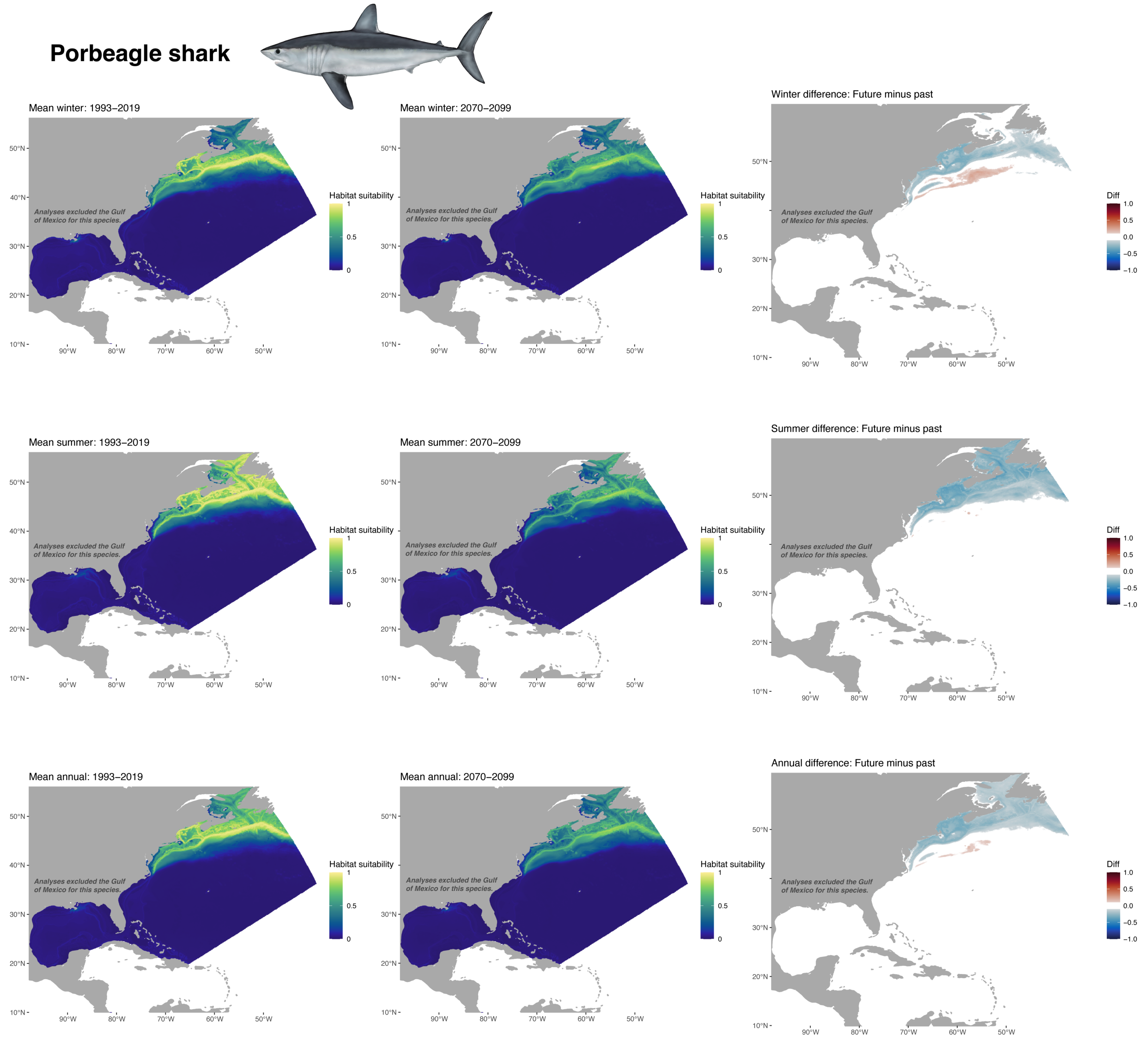

Figure S8: **Predicted change in species-specific habitat demonstrate significant spatial and seasonal variability.** Model predicted habitat suitability for porbeagle shark during summer (top row), winter (middle row) and the annual average (bottom row) for the current (left column) and future periods (middle column) and the expected change (right column). These predictions use the GFDL downscaled climate model. Note that ±10% change in habitat suitability in the right column is masked (white) to improve visual clarity for more substantial expected changes.

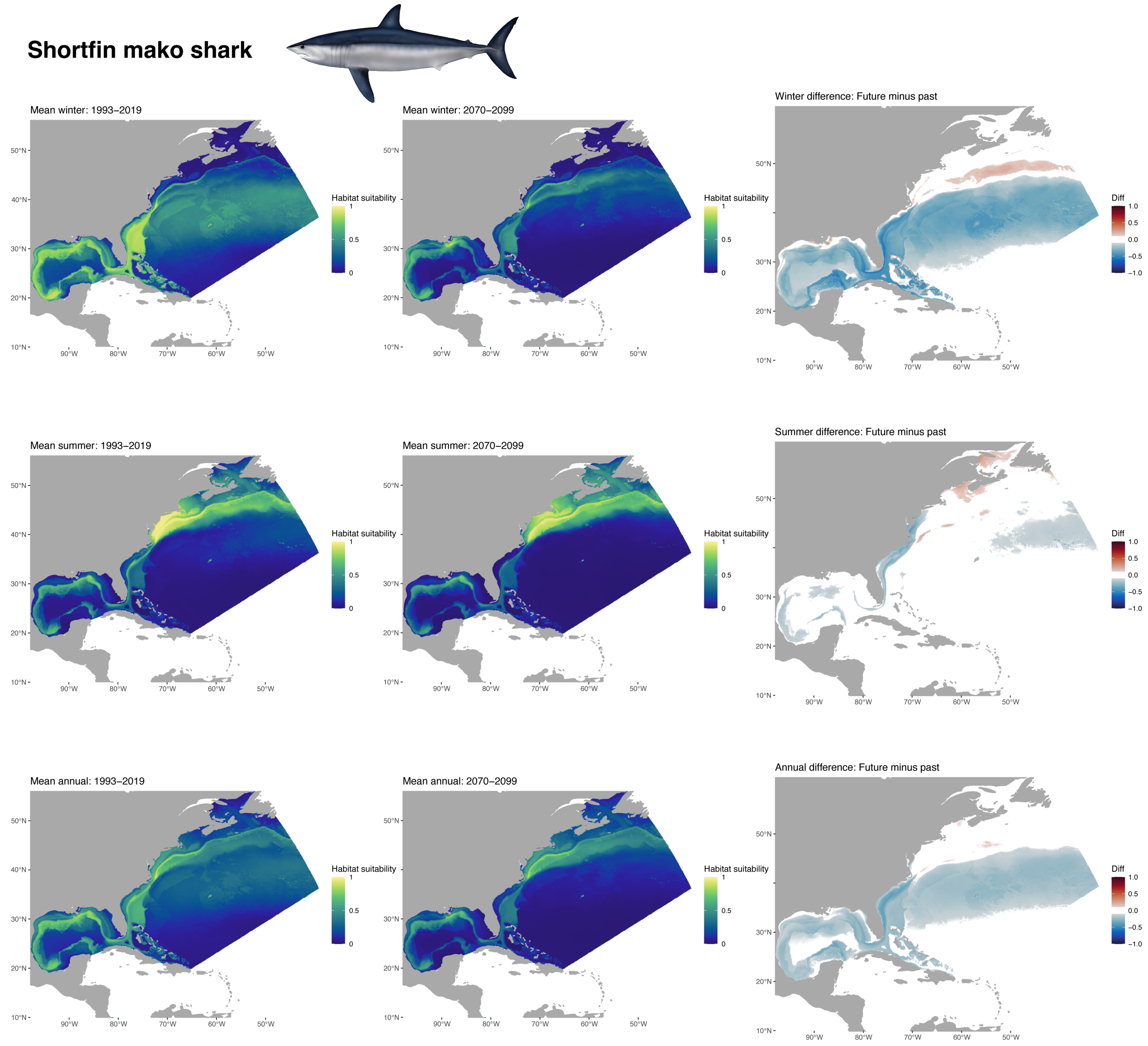

Figure S9: **Predicted change in species-specific habitat demonstrate significant spatial and seasonal variability.** Model predicted habitat suitability for shortfin mako shark during summer (top row), winter (middle row) and the annual average (bottom row) for the current (left column) and future periods (middle column) and the expected change (right column). These predictions use the GFDL downscaled climate model. Note that ±10% change in habitat suitability in the right column is masked (white) to improve visual clarity for more substantial expected changes.

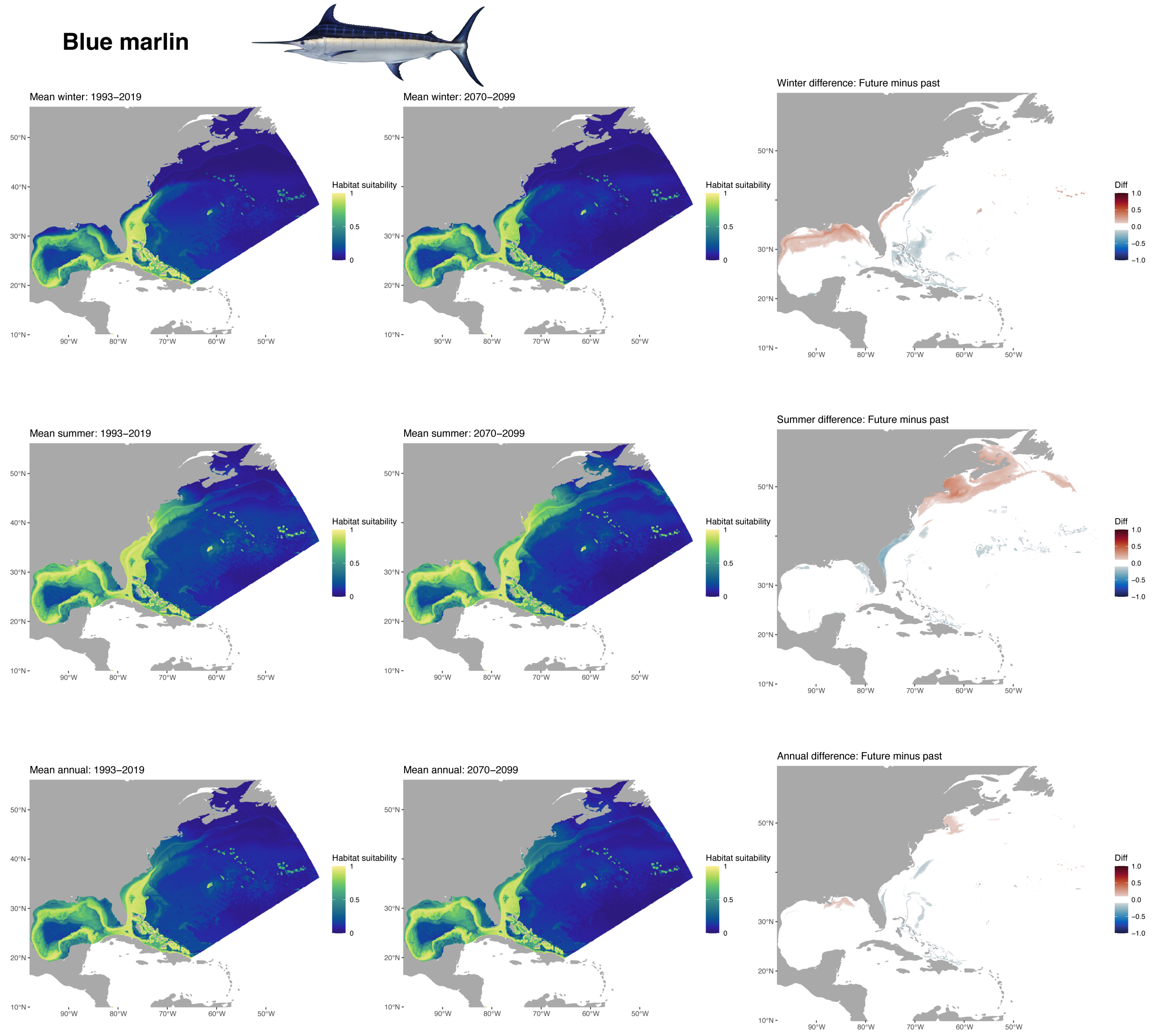

Figure S10: **Predicted change in species-specific habitat demonstrate significant spatial and seasonal variability.** Model predicted habitat suitability for Atlantic blue marlin during summer (top row), winter (middle row) and the annual average (bottom row) for the current (left column) and future periods (middle column) and the expected change (right column). These predictions use the GFDL downscaled climate model. Note that ±10% change in habitat suitability in the right column is masked (white) to improve visual clarity for more substantial expected changes.

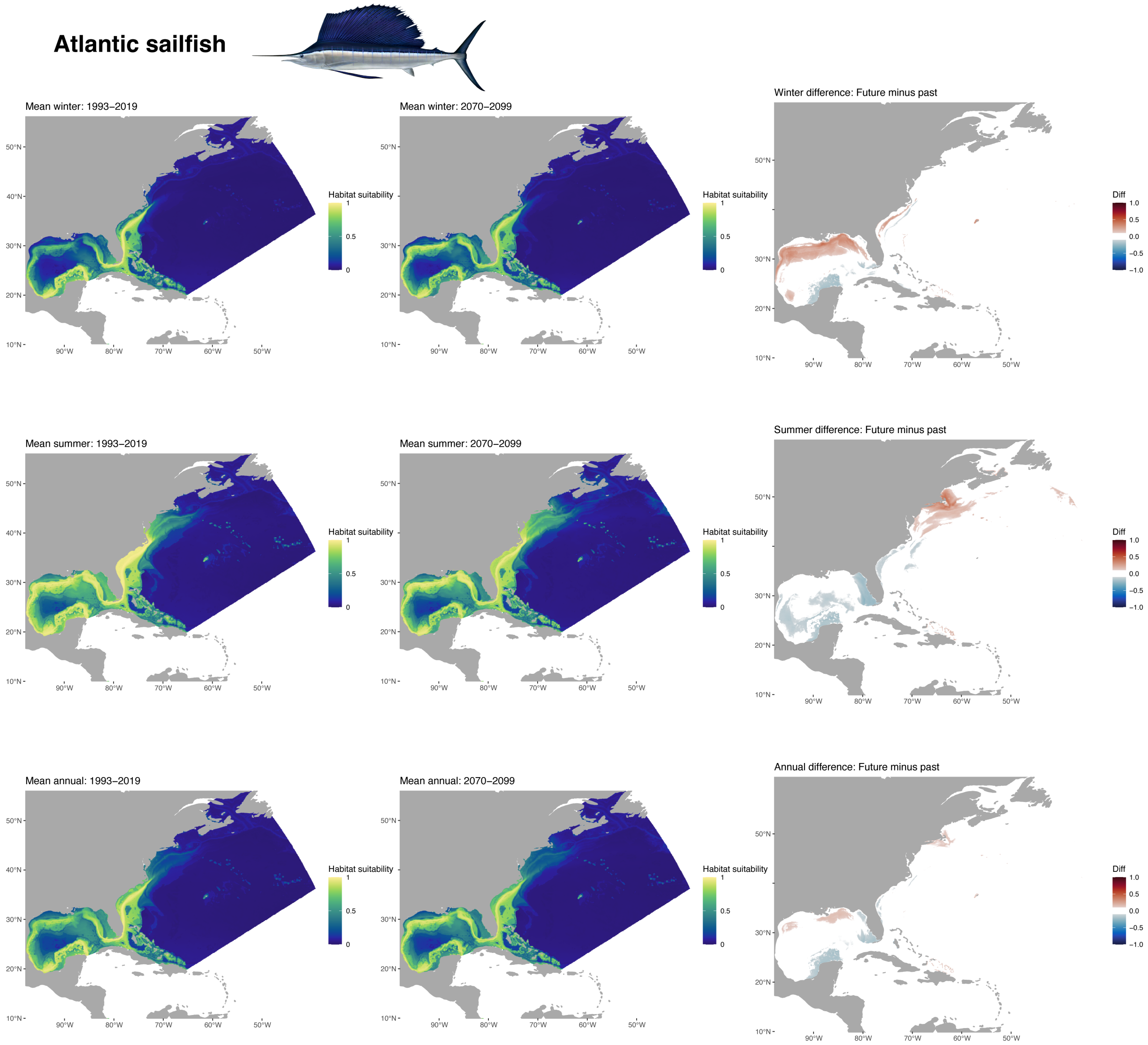

Figure S11: **Predicted change in species-specific habitat demonstrate significant spatial and seasonal variability.** Model predicted habitat suitability for Atlantic sailfish during summer (top row), winter (middle row) and the annual average (bottom row) for the current (left column) and future periods (middle column) and the expected change (right column). These predictions use the GFDL downscaled climate model. Note that ±10% change in habitat suitability in the right column is masked (white) to improve visual clarity for more substantial expected changes.

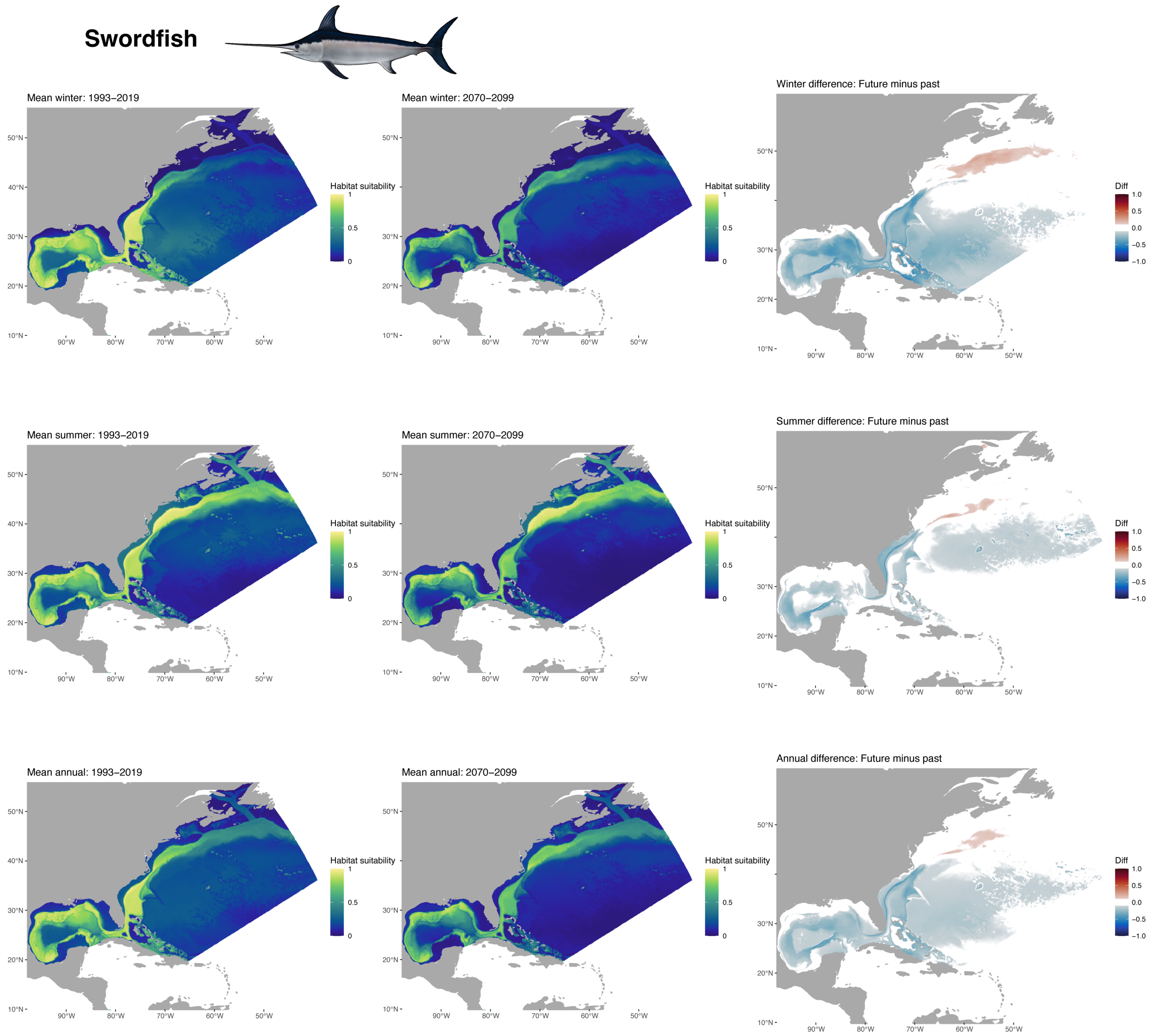

Figure S12: **Predicted change in species-specific habitat demonstrate significant spatial and seasonal variability.** Model predicted habitat suitability for swordfish during summer (top row), winter (middle row) and the annual average (bottom row) for the current (left column) and future periods (middle column) and the expected change (right column). These predictions use the GFDL downscaled climate model. Note that ±10% change in habitat suitability in the right column is masked (white) to improve visual clarity for more substantial expected changes.

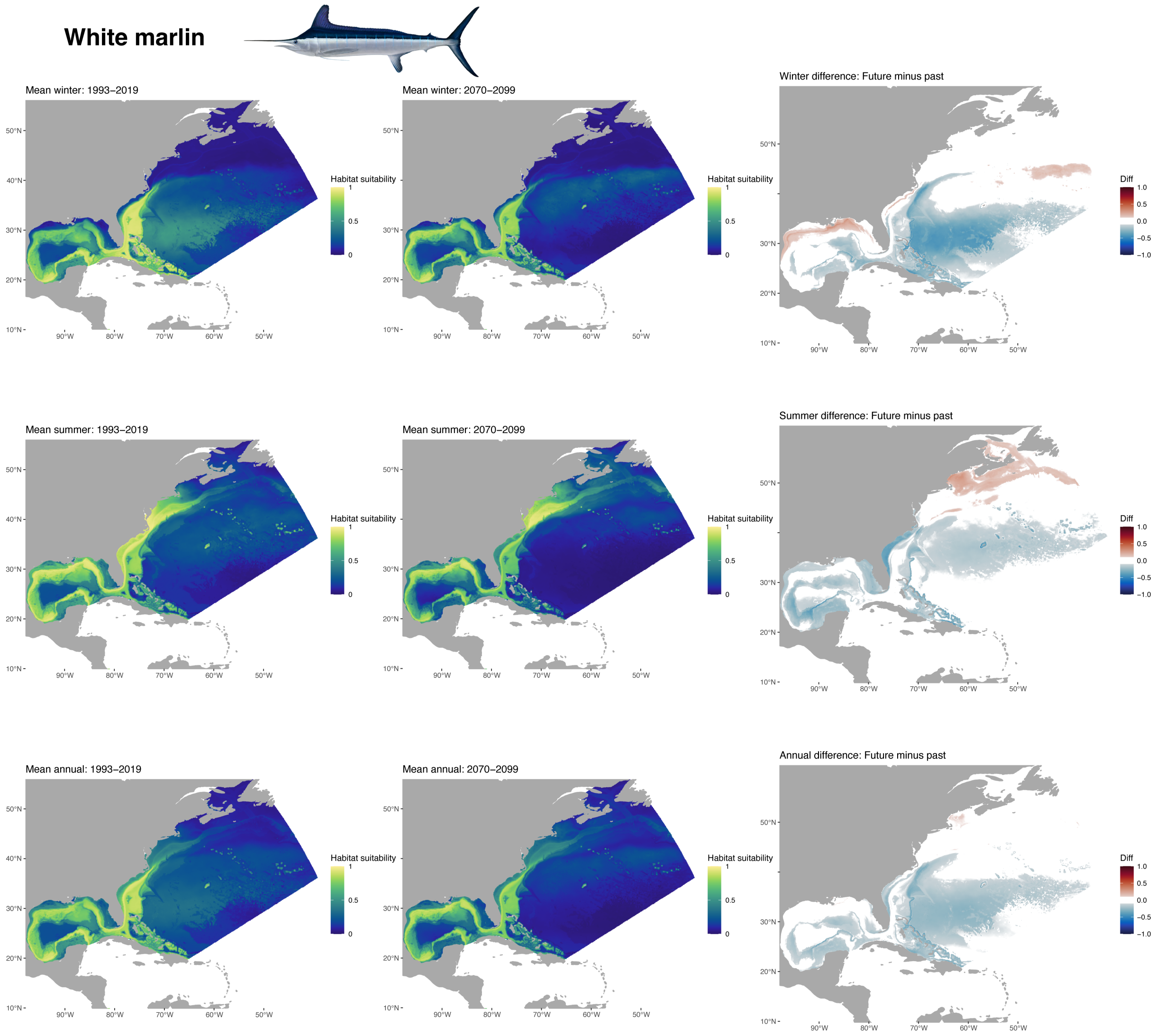

Figure S13: **Predicted change in species-specific habitat demonstrate significant spatial and seasonal variability.** Model predicted habitat suitability for white marlin during summer (top row), winter (middle row) and the annual average (bottom row) for the current (left column) and future periods (middle column) and the expected change (right column). These predictions use the GFDL downscaled climate model. Note that ±10% change in habitat suitability in the right column is masked (white) to improve visual clarity for more substantial expected changes.

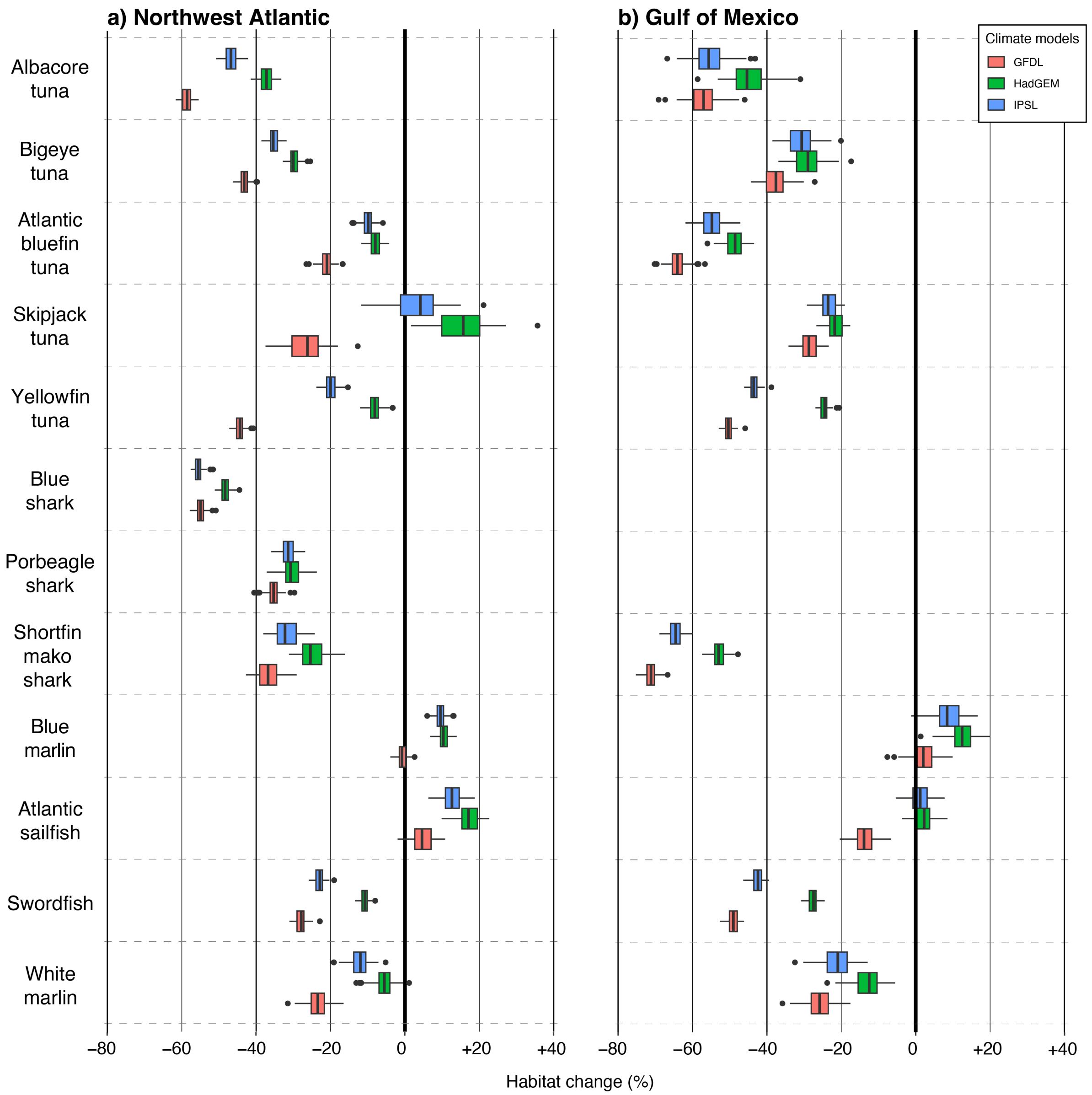

Figure S14: **Climate models generally agree on magnitude of expected habitat change.** Overall habitat change in the Northwest Atlantic (a) and Gulf of Mexico (b) for all bootstrap iterations (n=100) and downscaled global climate models (n=3, see Methods). Percentage values represent future change in suitable habitat area relative to the historical period.

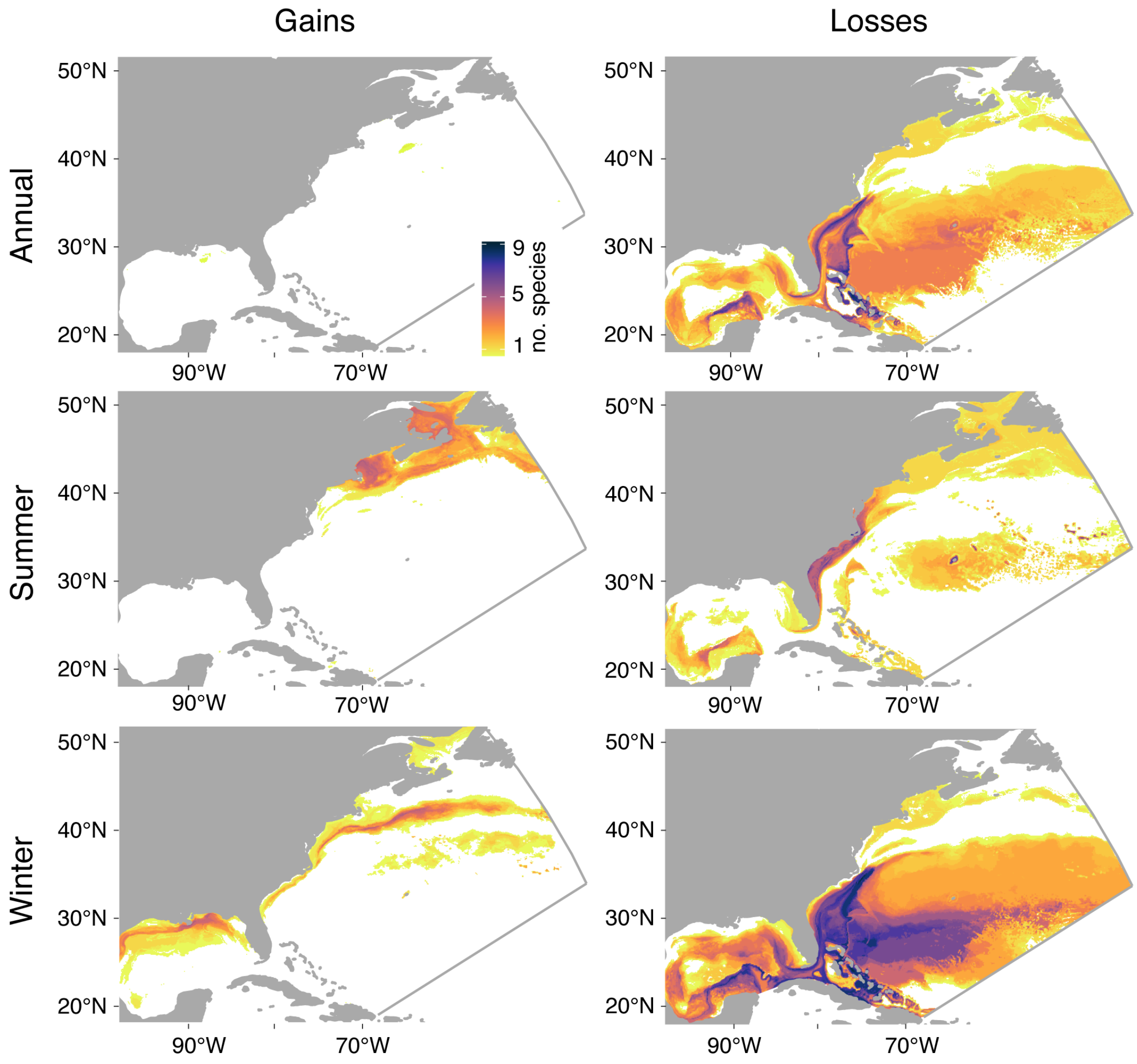

Figure S15: **Hotspots of multi-species habitat loss are more intense and widespread than expected habitat gains.** Hotspots of multi-species habitat gains (left) and losses (right) for annual (top) and seasonal (middle, bottom) averages. Each cell represents the number of species for which habitat suitability increased (gains) or decreased (losses) between the future and contemporary periods. Only cells containing >|20|% change in suitability for each species are included here. Species counts are averaged over the three downscaled global climate models used to project future habitat suitability. Downscaled global climate model domain is shown in gray outline.

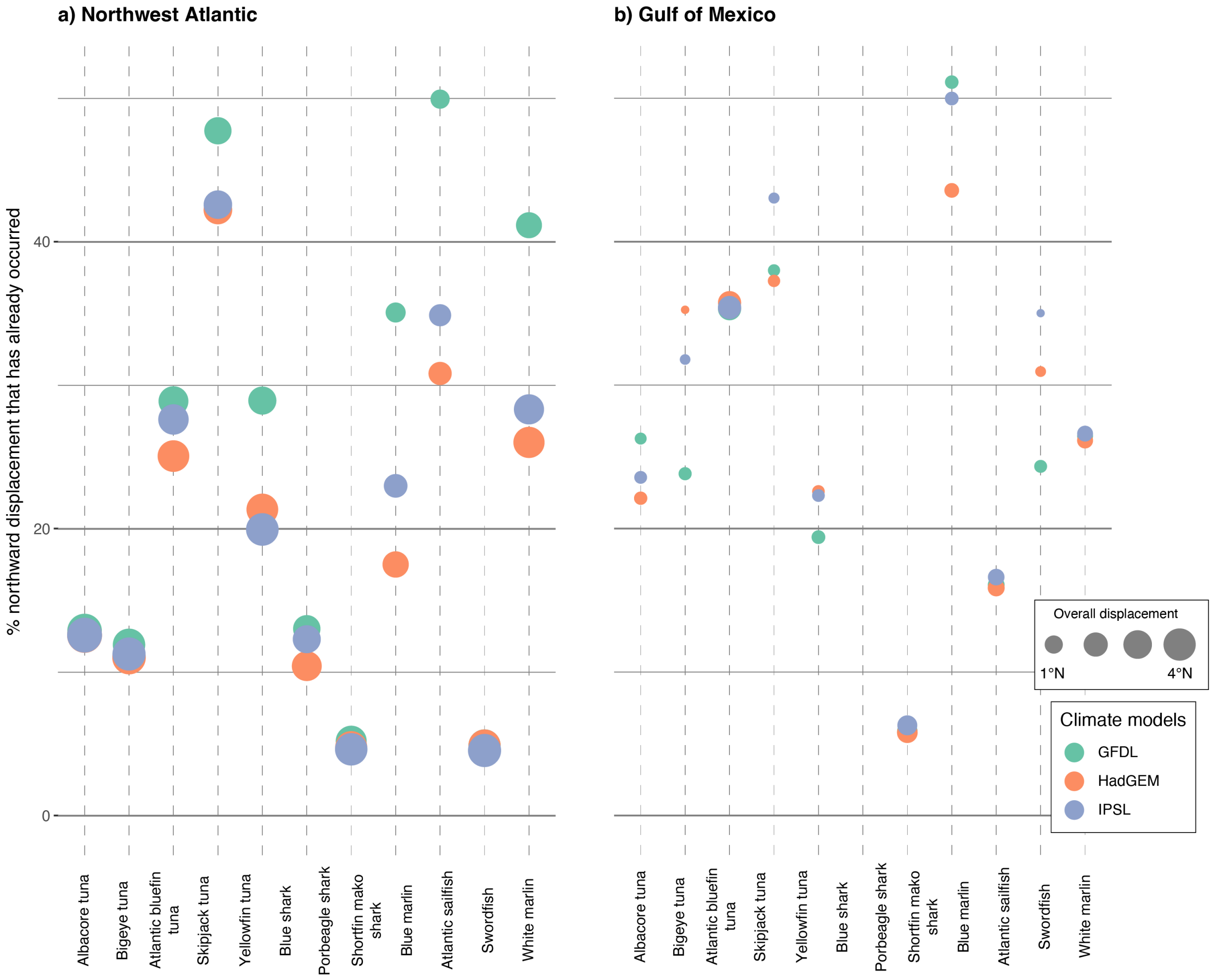

Figure S16: **Climate impacts are already apparent for some species.** The impacts of climate change that have already occurred, expressed as percentage of overall northward displacement expected by the end of the century (latitudinal anomaly of center-of-gravity) that has already occurred by the end of the contemporary period. Point sizes are scaled relative to the overall expected displacement. Note that porbeagle shark is removed from both panels.

Table S1: Summary of sample sizes and model evaluation metrics. Raw ICCAT marker tag (N_{marker}) and pelagic longline observer program ($N_{observer}$) datasets were quality controlled, subset to the spatial (North Atlantic) and temporal (1993-2019) extents of interest and combined to create a pooled model training dataset (N_{train}). R^2=percent explained deviance, AUC=Area Under the Receiver Operating Characteristic Curve, TSS=True Skill Statistic. See Methods for full explanation of these metrics. Model evaluation statistics were derived from 10-fold cross validation. Higher values indicate better model performance. *Note that white marlin likely represents both white marlin and roundscale spearfish (*Tetrapturus georgii*) given historical issues with species identification (78).

Common name	Scientific name	N_{marker}	$N_{observer}$	N_{train}	R2	AUC	TSS	Accuracy
Albacore tuna	*Thunnus alalunga*	1,048	4,801	5,016	0.55	0.94	0.74	0.86
Bigeye tuna	*Thunnus obesus*	4,419	6,904	9,800	0.48	0.92	0.68	0.84
Atl Bluefin tuna	*Thunnus thynnus*	10,061	2,611	7,602	0.70	0.97	0.84	0.92
Skipjack tuna	*Katsuwonus pelamis*	3,349	2,674	4,535	0.69	0.96	0.83	0.91
Yellowfin tuna	*Thunnus albacares*	5,697	14,799	17,535	0.63	0.96	0.80	0.90
Blue shark	*Prionace glauca*	54,876	8,126	39,100	0.62	0.95	0.78	0.89
Porbeagle shark	*Lamna nasus*	1,493	628	1,746	0.78	0.97	0.87	0.93
Shortfin mako shark	*Isurus oxyrinchus*	8,866	4,118	9,496	0.64	0.96	0.79	0.89
Blue marlin	*Makaira nigricans*	16,384	3,608	14,798	0.57	0.94	0.76	0.88
Atl Sailfish	*Istiophorus platypterus*	21,324	2,501	12,432	0.71	0.97	0.84	0.92
Swordfish	*Xiphias gladius*	12,022	18,971	24,616	0.56	0.94	0.76	0.88
White marlin*	*Kajikia albida*	14,082	5,470	12,927	0.59	0.95	0.77	0.88

Table S2: Relative importance of each environmental covariate in species-specific models.

		bathymetry	sst	ssh	sss	rugosity
**Tunas**	**Albacore tuna**	36.9	27.2	20.1	12.3	3.5
	**Bigeye tuna**	51.1	19.8	13.8	10.1	5.1
	**Atl bluefin tuna**	61.1	20.2	11.5	4.8	2.4
	**Skipjack tuna**	65.4	14.6	14.2	2.3	3.5
	**Yellowfin tuna**	67.5	12.0	8.7	6.6	5.3
**Sharks**	**Blue shark**	16.4	21.9	9.6	51.5	0.6
	**Porbeagle shark**	3.0	8.0	50.3	35.9	2.8
	**Shortfin mako shark**	18.8	20.9	44.7	13.3	2.4
**Billfish**	**Blue marlin**	69.1	18.3	4.3	2.2	6.1
	**Atlantic sailfish**	80.3	11.6	4.3	1.3	2.5
	**Swordfish**	58.3	15.2	11.2	12.7	2.6
	**White marlin**	64.2	21.7	7.6	4.0	2.4

REFERENCES AND NOTES

1. S. C. Doney, M. Ruckelshaus, J. Emmett Duffy, J. P. Barry, F. Chan, C. A. English, H. M. Galindo, J. M. Grebmeier, A. B. Hollowed, N. Knowlton, J. Polovina, N. N. Rabalais, W. J. Sydeman, L. D. Talley, Climate change impacts on marine ecosystems. *Ann. Rev. Mar. Sci.* **4**, 11–37 (2012).

2. C. H. Lam, B. Galuardi, A. Mendillo, E. Chandler, M. E. Lutcavage, Sailfish migrations connect productive coastal areas in the West Atlantic Ocean. *Sci. Rep.* **6**, 38163 (2016).

3. IPCC, Climate Change 2022: Impacts, Adaptation, and Vulnerability. Contribution of Working Group II to the Sixth Assessment Report of the Intergovernmental Panel on Climate Change, *Tech. Rep.* (Cambridge Univ. Press, 2022).

4. FAO, The State of the World Fisheries, Food and Agricultural Organization of the United Nations, *Tech. Rep.* (2016).

5. E. H. Allison, A. L. Perry, M. C. Badjeck, W. Neil Adger, K. Brown, D. Conway, A. S. Halls, G. M. Pilling, J. D. Reynolds, N. L. Andrew, N. K. Dulvy, Vulnerability of national economies to the impacts of climate change on fisheries. *Fish Fish.* **10**, 173–196 (2009).

6. M.-C. Badjeck, E. H. Allison, A. S. Halls, N. K. Dulvy, Impacts of climate variability and change on fishery-based livelihoods. *Mar. Policy* **34**, 375–383 (2010).

7. NMFS, Current Fishery Statistics No. 2016, Fisheries of the United States, *Tech. Rep.* (National Marine Fisheries Service, Office of Science and Technology, 2016).

8. J. S. Link, R. Griffis, S. Busch, (eds.) NOAA Fisheries Climate Science Strategy. *U.S. Dept. of Commerce, NOAA Technical Memorandum* NMFS-F/SPO, -70 (2015).

9. J. S. Link, J. A. Nye, J. A. Hare, Guidelines for incorporating fish distribution shifts into a fisheries management context. *Fish Fish.* **12**, 461–469 (2011).

10. M. L. Pinsky, A. Fredston, A stark future for ocean life. *Science* **376**, 452–453 (2022).

11. J. A. Smith, D. Tommasi, H. Welch, E. L. Hazen, J. Sweeney, S. Brodie, B. Muhling, S. M. Stohs, M.

G. Jacox, Comparing dynamic and static time-area closures for Bycatch mitigation: A management strategy evaluation of a swordfish fishery. *Front. Mar. Sci.* **8**, 1–19 (2021).

12. N. Queiroz, N.E. Humphries, A. Couto, M. Vedor, I. da Costa, A.M.M. Sequeira, G. Mucientes, A.M. Santos, F.J. Abascal, D.L. Abercrombie, K. Abrantes, D. Acuña-Marrero, A.S. Afonso, P. Afonso, D. Anders, G. Araujo, R. Arauz, P. Bach, A. Barnett, D. Bernal, M.L. Berumen, S. Bessudo Lion, N.P.A. Bezerra, A.V. Blaison, B.A. Block, M.E. Bond, R. Bonfil, R.W. Bradford, C.D. Braun, E.J. Brooks, A. Brooks, J. Brown, B.D. Bruce, M.E. Byrne, S.E. Campana, A.B. Carlisle, D.D. Chapman, T.K. Chapple, J. Chisholm, C.R. Clarke, E.G. Clua, J.E.M. Cochran, E.C. Crochelet, L. Dagorn, R. Daly, D.D. Cortés, T.K. Doyle, M. Drew, C.A.J. Duffy, T. Erikson, E. Espinoza, L.C. Ferreira, F. Ferretti, J.D. Filmalter, G.C. Fischer, R. Fitzpatrick, J. Fontes, F. Forget, M. Fowler, M.P. Francis, A.J. Gallagher, E. Gennari, S.D. Goldsworthy, M.J. Gollock, J.R. Green, J.A. Gustafson, T.L. Guttridge, H.M. Guzman, N. Hammerschlag, L. Harman, F.H.V. Hazin, M. Heard, A.R. Hearn, J.C. Holdsworth, B.J. Holmes, L.A. Howey, M. Hoyos, R.E. Hueter, N.E. Hussey, C. Huveneers, D.T. Irion, D.M.P. Jacoby, O.J.D. Jewell, R. Johnson, L.K.B. Jordan, S.J. Jorgensen, W. Joyce, C.A. Keating Daly, J.T. Ketchum, A.P. Klimley, A.A. Kock, P. Koen, F. Ladino, F.O. Lana, J.S.E. Lea, F. Llewellyn, W.S. Lyon, A. MacDonnell, B.C.L. Macena, H. Marshall, J.D. McAllister, R. McAuley, M.A. Meÿer, J.J. Morris, E.R. Nelson, Y.P. Papastamatiou, T.A. Patterson, C. Peñaherrera-Palma, J.G. Pepperell, S.J. Pierce, F. Poisson, L.M. Quintero, A.J. Richardson, P.J. Rogers, C.A. Rohner, D.R.L. Rowat, M. Samoilys, J.M. Semmens, M. Sheaves, G. Shillinger, M. Shivji, S. Singh, G.B. Skomal, M.J. Smale, L.B. Snyders, G. Soler, M. Soria, K.M. Stehfest, J.D. Stevens, S.R. Thorrold, M.T. Tolotti, A. Towner, P. Travassos, J.P. Tyminski, F. Vandeperre, J.J. Vaudo, Y.Y. Watanabe, S.B. Weber, B.M. Wetherbee, T.D. White, S. Williams, P.M. Zárate, R. Harcourt, G.C. Hays, M.G. Meekan, M. Thums, X. Irigoien, V.M. Eguiluz, C.M. Duarte, L.L. Sousa, S.J. Simpson, E.J. Southall, D.W. Sims, Global spatial risk assessment of sharks under the footprint of fisheries. *Nature* **572**, 461–466 (2019).

13. E. Sala, J. Mayorga, D. Bradley, R. B. Cabral, T. B. Atwood, A. Auber, W. Cheung, C. Costello, F. Ferretti, A. M. Friedlander, S. D. Gaines, C. Garilao, W. Goodell, B. S. Halpern, A. Hinson, K. Kaschner, K. Kesner-Reyes, F. Leprieur, J. McGowan, L. E. Morgan, D. Mouillot, J. Palacios-Abrantes, H. P. Possingham, K. D. Rechberger, B. Worm, J. Lubchenco, Protecting the global ocean for biodiversity, food and climate. *Nature* **592**, 397–402 (2021).

14. K. M. Kleisner, M. J. Fogarty, S. McGee, A. Barnett, P. Fratantoni, J. Greene, J. A. Hare, S. M. Lucey, C. McGuire, J. Odell, V. S. Saba, L. Smith, K. J. Weaver, M. L. Pinsky, The effects of sub-regional climate velocity on the distribution and spatial extent of marine species assemblages. *PLOS ONE* **11**, e0149220 (2016).

15. J. A. Nye, J. S. Link, J. A. Hare, W. J. Overholtz, Changing spatial distribution of fish stocks in relation to climate and population size on the Northeast United States continental shelf. *Mar. Ecol. Prog. Ser.* **393**, 111–129 (2009).

16. A. J. Pershing, K. E. Mills, N. R. Record, K. Stamieszkin, K. V. Wurtzell, C. J. Byron, D. Fitzpatrick, W. J. Golet, E. Koob, Evaluating trophic cascades as drivers of regime shifts in different ocean ecosystems. *Philos. Trans. R. Soc. B. Biol. Sci.* **370**, 20130265 (2015).

17. R. L. Selden, R. D. Batt, V. S. Saba, M. L. Pinsky, Diversity in thermal affinity among key piscivores buffers impacts of ocean warming on predator–prey interactions. *Glob. Chang. Biol.* **24**, 117–131 (2018).

18. V. S. Saba, S. M. Griffies, W. G. Anderson, M. Winton, M. A. Alexander, T. L. Delworth, J. A. Hare, M. J. Harrison, A. Rosati, G. A. Vecchi, R. Zhang, Enhanced warming of the Northwest Atlantic Ocean under climate change. *J. Geophys. Res. Oceans* **120**, 118–132 (2016).

19. Y. Liu, S.-K. Lee, D. B. Enfield, B. A. Muhling, J. T. Lamkin, F. E. Muller-Karger, M. A. Roffer, Potential impact of climate change on the Intra-Americas Sea: Part-1. A dynamic downscaling of the CMIP5 model projections. *J. Mar. Syst.* **148**, 56–69 (2015).

20. M. A. Alexander, S. I. Shin, J. D. Scott, E. Curchitser, C. Stock, The response of the Northwest Atlantic Ocean to climate change. *J. Climate* **33**, 405–428 (2020).

21. E. C. Oliver, J. A. Benthuysen, S. Darmaraki, M. G. Donat, A. J. Hobday, N. J. Holbrook, R. W. Schlegel, A. Sen Gupta, Marine heatwaves. *Ann. Rev. Mar. Sci.* **13**, 313–342 (2021).

22. M. G. Burgess, S. L. Becker, R. E. Langendorf, A. Fredston, C. M. Brooks, Climate change scenarios in fisheries and aquatic conservation research. *ICES J. Mar. Sci.* **80**, 1163–1178 (2023).

23. B. Abrahms, N. H. Carter, T. J. Clark-Wolf, K. M. Gaynor, E. Johansson, A. McInturff, A. C. Nisi, K. Rafiq, L. West, Climate change as a global amplifier of human–wildlife conflict. *Nat. Clim. Chang.* **13**, 224–234 (2023).

24. E. A. Fulton, Interesting times: Winners, losers, and system shifts under climate change around Australia. *ICES J. Mar. Sci.* **68**, 1329–1342 (2011).

25. E. L. Hazen, S. Jorgensen, R. R. Rykaczewski, S. J. Bograd, D. G. Foley, I. D. Jonsen, S. A. Shaffer, J. P. Dunne, D. P. Costa, L. B. Crowder, B. A. Block, Predicted habitat shifts of Pacific top predators in a changing climate. *Nat. Clim. Chang.* **3**, 234–238 (2013).

26. J. A. Hare, W. E. Morrison, M. W. Nelson, M. M. Stachura, E. J. Teeters, R. B. Griffis, M. A. Alexander, J. D. Scott, L. Alade, R. J. Bell, A. S. Chute, K. L. Curti, T. H. Curtis, D. Kircheis, J. F. Kocik, S. M. Lucey, C. T. McCandless, L. M. Milke, D. E. Richardson, E. Robillard, H. J. Walsh, M. C. McManus, K. E. Marancik, C. A. Griswold, A vulnerability assessment of fish and invertebrates to climate change on the northeast u.s. continental shelf. *PLOS ONE* **11**, e0146756 (2016).

27. G. N. Somero, The physiology of climate change: How potentials for acclimatization and genetic adaptation will determine 'winners' and 'losers'. *J. Exp. Biol.* **213**, 912–920 (2010).

28. J. M. Grady, B. S. Maitner, A. S. Winter, K. Kaschner, D. P. Tittensor, S. Record, F. A. Smith, A. M. Wilson, A. I. Dell, P. L. Zarnetske, H. J. Wearing, B. Alfaro, J. H. Brown, Metabolic asymmetry and the global diversity of marine predators. *Science* **363**, eaat4220 (2019).

29. S. L. H. Teo, A. Boustany, H. Dewar, M. J. W. Stokesbury, K. C. Weng, S. Beemer, A. C. Seitz, C. J. Farwell, E. D. Prince, B. A. Block, Annual migrations, diving behavior, and thermal biology of Atlantic bluefin tuna, *Thunnus thynnus*, on their Gulf of Mexico breeding grounds. *Mar. Biol.* **151**, 1–18 (2007).

30. B. A. Muhling, S. K. Lee, J. T. Lamkin, Y. Liu, Predicting the effects of climate change on bluefin tuna (*Thunnus thynnus*) spawning habitat in the Gulf of Mexico. *ICES J. Mar. Sci.* **68**, 1051–1062 (2011).

31. E. L. Hazen, A. B. Carlisle, S. G. Wilson, J. E. Ganong, M. R. Castleton, R. J. Schallert, M. J. Stokesbury, S. J. Bograd, B. A. Block, Corrigendum: Quantifying overlap between the Deepwater Horizon oil spill and predicted bluefin tuna spawning habitat in the Gulf of Mexico. *Sci. Rep.* **6**, 36689

(2016).

32. W. W. Cheung, R. Watson, D. Pauly, Signature of ocean warming in global fisheries catch. *Nature* **497**, 365–368 (2013).

33. D. P. Crear, T. H. Curtis, C. P. Hutt, Y. W. Lee, Climate-influenced shifts in a highly migratory species recreational fishery. *Fish. Oceanogr.* **32**, 327–340 (2023).

34. S. Dueri, P. Guillotreau, R. Jiménez-Toribio, R. Oliveros-Ramos, L. Bopp, O. Maury, Food security or economic profitability? Projecting the effects of climate and socioeconomic changes on global skipjack tuna fisheries under three management strategies. *Glob. Environ. Chang.* **41**, 1–12 (2016).

35. L. A. Rogers, R. Griffin, T. Young, E. Fuller, K. St. Martin, M. L. Pinsky, Shifting habitats expose fishing communities to risk under climate change. *Nature* **9**, 512–516 (2019).

36. L. M. Robinson, A. J. Hobday, H. P. Possingham, A. J. Richardson, Trailing edges projected to move faster than leading edges for large pelagic fish habitats under climate change. *Deep Sea Res. II Top. Stud. Oceanogr.* **113**, 225–234 (2015).

37. M. Barange, G. Merino, J. L. Blanchard, J. Scholtens, J. Harle, E. H. Allison, J. I. Allen, J. Holt, S. Jennings, Impacts of climate change on marine ecosystem production in societies dependent on fisheries. *Nat. Clim. Chang.* **4**, 211–216 (2014).

38. M. Erauskin-Extramiana, H. Arrizabalaga, A. J. Hobday, A. Cabré, L. Ibaibarriaga, I. Arregui, H. Murua, G. Chust, Large-scale distribution of tuna species in a warming ocean. *Glob. Chang. Biol.* **25**, 2043–2060 (2019).

39. N. Farchadi, M. G. Hinton, A. R. Thompson, Z.-Y. Yin, Modeling the dynamic habitats of mobile pelagic predators (*Makaira nigricans* and *Istiompax indica*) in the eastern Pacific Ocean. *Mar. Ecol. Prog. Ser.* **622**, 157–176 (2019).

40. J. J. Dale, S. Brodie, A. B. Carlisle, M. Castleton, E. L. Hazen, S. J. Bograd, B. A. Block, Global habitat loss of a highly migratory predator, the blue marlin (*Makaira nigricans*). *Divers. Distrib.* **28**, 2020–2034 (2022).

41. M. A. Karp, S. Brodie, J. A. Smith, K. Richerson, R. L. Selden, O. R. Liu, B. A. Muhling, J. F. Samhouri, L. A. Barnett, E. L. Hazen, D. Ovando, J. Fiechter, M. G. Jacox, M. Pozo Buil, Projecting species distributions using fishery-dependent data. *Fish Fish.* **24**, 71–92 (2023).

42. N. J. Isaac, M. A. Jarzyna, P. Keil, L. I. Dambly, P. H. Boersch-Supan, E. Browning, S. N. Freeman, N. Golding, G. Guillera-Arroita, P. A. Henrys, S. Jarvis, J. Lahoz-Monfort, J. Pagel, O. L. Pescott, R. Schmucki, E. G. Simmonds, R. B. O'Hara, Data integration for large-scale models of species distributions. *Trends Ecol. Evol.* **35**, 56–67 (2020).

43. P. Lehodey, I. Senina, B. Calmettes, J. Hampton, S. Nicol, Modelling the impact of climate change on Pacific skipjack tuna population and fisheries. *Clim. Chang.* **119**, 95–109 (2013).

44. E. S. Poloczanska, C. J. Brown, W. J. Sydeman, W. Kiessling, D. S. Schoeman, P. J. Moore, K. Brander, J. F. Bruno, L. B. Buckley, M. T. Burrows, C. M. Duarte, B. S. Halpern, J. Holding, C. V. Kappel, M. I. O'Connor, J. M. Pandolfi, C. Parmesan, F. Schwing, S. A. Thompson, A. J. Richardson, Global imprint of climate change on marine life. *Nat. Clim. Chang.* **3**, 919–925 (2013).

45. E. L. Hazen, K. L. Scales, S. M. Maxwell, D. K. Briscoe, H. Welch, S. J. Bograd, H. Bailey, S. R. Benson, T. Eguchi, H. Dewar, S. Kohin, D. P. Costa, L. B. Crowder, R. L. Lewison, A dynamic ocean management tool to reduce bycatch and support sustainable fisheries. *Sci. Adv.* **4**, eaar3001 (2018).

46. D. P. Crear, T. H. Curtis, S. J. Durkee, J. K. Carlson, Highly migratory species predictive spatial modeling (PRiSM): An analytical framework for assessing the performance of spatial fisheries management. *Mar. Biol.* **168**, 148 (2021).

47. C. D. Braun, P. Gaube, P. Afonso, J. Fontes, G. B. Skomal, S. R. Thorrold, Assimilating electronic tagging, oceanographic modelling, and fisheries data to estimate movements and connectivity of swordfish in the North Atlantic. *ICES J. Mar. Sci.* **76**, 2305–2317 (2019).

48. M. C. Arostegui, P. Gaube, P. A. Woodworth-Jefcoats, D. R. Kobayashi, C. D. Braun, Anticyclonic eddies aggregate pelagic predators in a subtropical gyre. *Nature* **609**, 535–540 (2022).

49. K. D. Hyrenbach, C. Keiper, S. G. Allen, D. G. Ainley, D. J. Anderson, Use of marine sanctuaries by far-ranging predators: Commuting flights to the California Current System by breeding Hawaiian

albatrosses. *Fish. Oceanogr.* **15**, 95–103 (2006).

50. R. Lewison, A. J. Hobday, S. Maxwell, E. Hazen, J. R. Hartog, D. C. Dunn, D. Briscoe, S. Fossette, C. E. O'Keefe, M. Barnes, M. Abecassis, S. Bograd, N. David Bethoney, H. Bailey, D. Wiley, S. Andrews, L. Hazen, L. B. Crowder, Dynamic ocean management: Identifying the critical ingredients of dynamic approaches to ocean resource management. *Bioscience* **65**, 486–498 (2015).

51. S. M. Maxwell, K. M. Gjerde, M. G. Conners, L. B. Crowder, Mobile protected areas for biodiversity on the high seas. *Science* **367**, 252–254 (2020).

52. J. A. Santora, N. J. Mantua, I. D. Schroeder, J. C. Field, E. L. Hazen, S. J. Bograd, W. J. Sydeman, B. K. Wells, J. Calambokidis, L. Saez, D. Lawson, K. A. Forney, Habitat compression and ecosystem shifts as potential links between marine heatwave and record whale entanglements. *Nat. Commun.* **11**, 536 (2020).

53. B. Abrahms, Human-wildlife conflict under climate change. *Science* **373**, 484–485 (2021).

54. L. R. Beerkircher, E. Cortés, M. Shivji, Characteristics of shark bycatch observed on pelagic longlines off the Southeastern United States, 1992–2000. **64**, 40–49 (2002).

55. M. Barbet-Massin, F. Jiguet, C. H. Albert, W. Thuiller, Selecting pseudo-absences for species distribution models: How, where and how many? *Methods Ecol. Evol.* **3**, 327–338 (2012).

56. E. L. Hazen, B. Abrahms, S. Brodie, G. Carroll, H. Welch, S. J. Bograd, Where did they not go? Considerations for generating pseudo-absences for telemetry-based habitat models. *Mov. Ecol.* **9**, 5 (2021).

57. J. Pinti, M. Shatley, A. Carlisle, B. A. Block, M. J. Oliver, Using pseudo-absence models to test for environmental selection in marine movement ecology: The importance of sample size and selection strength. *Mov. Ecol.* **10**, 60 (2022).

58. C. D. Braun, M. C. Arostegui, N. Farchadi, M. A. Alexander, P. Afonso, A. Allyn, S. J. Bograd, S. J. Brodie, D. P. Crear, E. Culhane, T. H. Curtis, E. L. Hazen, A. Kerney, N. Lezama-Ochoa, K. E. Mills, D. Pugh, N. Queiroz, J. D. Scott, G. B. Skomal, D. W. Sims, S. R. Thorrold, H. Welch, R. Young-

Morse, R. Lewison, Building use-inspired species distribution models: Using multiple data types to examine and improve model performance. *Ecol. Appl.* e2893 (2023).

59. S. Brodie, L. Litherland, J. Stewart, H. T. Schilling, J. G. Pepperell, I. M. Suthers, Citizen science records describe the distribution and migratory behaviour of a piscivorous predator, *Pomatomus saltatrix*. *ICES J. Mar. Sci.* **75**, 1573–1582 (2018).

60. J.-M. Lellouche, E. Greiner, O. Le Galloudec, G. Garric, C. Regnier, M. Drevillon, M. Benkiran, C.-E. Testut, R. Bourdalle-Badie, F. Gasparin, O. Hernandez, B. Levier, Y. Drilled, E. Remy, P.-Y. Le Traon, Recent updates to the Copernicus Marine Service global ocean monitoring and forecasting real-time 1⁄12° high-resolution system. *Ocean Science* **14**, 1093–1126 (2018).

61. A. F. Shchepetkin, J. C. McWilliams, The regional oceanic modeling system (ROMS): A split-explicit, free-surface, topography-following-coordinate oceanic model. *Ocean Model.* **9**, 347–404 (2005).

62. D. Kang, E. N. Curchitser, Gulf Stream eddy characteristics in a high-resolution ocean model. *J. Geophys. Res. Oceans* **118**, 4474–4487 (2013).

63. D. Brickman, M. A. Alexander, A. Pershing, J. D. Scott, Z. Wang, Projections of physical conditions in the Gulf of Maine in 2050. *Elem. Sci. Anth.* **9**, 00055 (2021).

64. S. A. Siedlecki, D. Pilcher, E. M. Howard, C. Deutsch, P. MacCready, E. L. Norton, H. Frenzel, J. Newton, R. A. Feely, S. R. Alin, Coastal processes modify projections of some climate-driven stressors in the California Current System. *Biogeosciences* **18**, 2871–2890 (2021).

65. H. Akima, A. Gebhardt, akima: Interpolation of irregularly and regularly spaced data. R package version 0.6–2 (2016).

66. M. R. Payne, M. Kudahl, G. H. Engelhard, M. A. Peck, J. K. Pinnegar, Climate risk to European fisheries and coastal communities. *Proc. Natl. Acad. Sci. U.S.A.* **118**, e2018086118 (2021).

67. J. Elith, C. H. Graham, R. P. Anderson, M. Dudík, S. Ferrier, A. Guisan, R. J. Hijmans, F. Huettmann, J. R. Leathwick, A. Lehmann, J. Li, L. G. Lohmann, B. A. Loiselle, G. Manion, C. Moritz, M. Nakamura, Y. Nakazawa, J. M. Overton, A. Townsend Peterson, S. J. Phillips, K. Richardson, R.

Scachetti-Pereira, R. E. Schapire, J. Soberón, S. Williams, M. S. Wisz, N. E. Zimmermann, Novel methods improve prediction of species' distributions from occurrence data. *Ecography* **29**, 129–151 (2006).

68. J. Elith, J. R. Leathwick, T. Hastie, A working guide to boosted regression trees. *J. Anim. Ecol.* **77**, 802–813 (2008).

69. B. Abrahms, H. Welch, S. Brodie, M. G. Jacox, E. A. Becker, S. J. Bograd, L. M. Irvine, D. M. Palacios, B. R. Mate, E. L. Hazen, Dynamic ensemble models to predict distributions and anthropogenic risk exposure for highly mobile species. *Diver. Distrib*. **25**, 1182–1193 (2019).

70. A. Norberg, N. Abrego, F. G. Blanchet, F. R. Adler, B. J. Anderson, J. Anttila, M. B. Araújo, T. Dallas, D. Dunson, J. Elith, S. D. Foster, R. Fox, J. Franklin, W. Godsoe, A. Guisan, B. O'Hara, N. A. Hill, R. D. Holt, F. K. Hui, M. Husby, J. A. Kålås, A. Lehikoinen, M. Luoto, H. K. Mod, G. Newell, I. Renner, T. Roslin, J. Soininen, W. Thuiller, J. Vanhatalo, D. Warton, M. White, N. E. Zimmermann, D. Gravel, O. Ovaskainen, A comprehensive evaluation of predictive performance of 33 species distribution models at species and community levels. *Ecol. Monogr*. **89**, e01370 (2019).

71. D. G. Altman, J. M. Bland, Diagnostic tests. 1: Sensitivity and specificity. *BMJ* **308**, 1552 (1994).

72. J. McHenry, H. Welch, S. E. Lester, V. Saba, Projecting marine species range shifts from only temperature can mask climate vulnerability. *Glob. Chang. Biol.* **25**, 4208–4221 (2019).

73. X. Robin, N. Turck, A. Hainard, N. Tiberti, F. Lisacek, J.-C. Sanchez, M. Müller, pROC: An open-source package for R and S+ to analyze and compare ROC curves. *BMC Bioinform.* **12**, 77 (2011).

74. S. Yalcin, S. J. Leroux, Diversity and suitability of existing methods and metrics for quantifying species range shifts. *Glob. Ecol. Biogeogr.* **26**, 609–624 (2017).

75. J. VanDerWal, L. Falconi, S. Januchowski, L. Shoo, C. Storlie, M. J. VanDerWal, Package 'SDMTools'. *R package* (2014).

76. D. Nychka, R. Furrer, J. Paige, S. Sain, M. D. Nychka, Package 'fields'; http://cran.r-project.org/web/packages/fields/fields.pdf (2015).

77. R. J. Hijmans, E. Williams, C. Vennes, M. R. J. Hijmans, Package ‘geosphere’. *Spherical Trigonometry* **1**, 1–45 (2017).

78. L. Beerkircher, F. Arocha, A. Barse, E. Prince, V. Restrepo, J. Serafy, M. Shivji, Effects of species misidentification on population assessment of overfished white marlin *Tetrapturus albidus* and roundscale spearfish *T. georgii*. *Endang. Species Res.* **9**, 81–89 (2010).
